# Enhanced degradation and defluorination of perfluorooctane sulfonate (PFOS) in tap water using gas-dispersed cold atmospheric plasma

**DOI:** 10.1038/s41598-026-57490-6

**Published:** 2026-06-13

**Authors:** Amit Kumar, Ysabel Huaccallo-Aguilar, Holger Kryk, Uwe Hampel, Sebastian Felix Reinecke

**Affiliations:** 1 Clean Water Technology Lab (CLEWATEC), Institute of Fluid Dynamics, Helmholtz Zentrum Dresden-Rossendorf, Bautzner Landstrasse 400, 01328 Dresden, Germany; 2 Technische Universität Dresden, Chair of Imaging Techniques in Energy and Process Engineering, 01062 Dresden, Germany

**Keywords:** Emerging contaminants, PFOS, Cold atmospheric plasma, Gas dispersion, Oxidative and reductive reactive species, Reaction pathways, Rapid degradation and partial defluorination, Chemistry, Engineering, Environmental sciences

## Abstract

Per- and polyfluoroalkyl substances (PFAS) are extremely persistent contaminants owing to the exceptional chemical stability of carbon–fluorine (C–F) bonds. Consequently, conventional wastewater treatments are largely ineffective, as they capture but fail to destroy PFAS, leading to the accumulation of concentrated wastes. In this study, we demonstrate that gas dispersion-assisted cold atmospheric plasma (CAP) enables rapid degradation and partial defluorination of perfluorooctane sulfonate (PFOS) in tap water. Operating under ambient conditions, CAP generates a rich mixture of oxidative and reductive reactive species, including solvated electrons and hydroxyl radicals, which are proposed to contribute to PFOS degradation and defluorination. Air gas dispersion enhances hydrodynamic mixing and enriches PFOS at the plasma-liquid interface, promoting interfacial microdischarges and concentrated short-lived reactive species that enhance oxidative and reductive degradation pathways. At high PFOS concentrations in tap water, gas dispersion-assisted CAP achieved 99.99% PFOS degradation with partial defluorination of 35%. With gas dispersion, degradation followed apparent first-order kinetics, with a rate constant of 0.42 1/min and a half-life of 1.6 min. In both conditions, with and without gas dispersion, analysis of measured transformation products (TPs) revealed stepwise degradation pathways of PFOS, with fluorine mass balance recoveries ranging from 31 to 106%. The lowest electrical energy per order (*EEO*) achieved was 39 kWh/m^3^/order. These results demonstrate the efficient degradation of PFOS, while the measured fluoride ion release confirms partial defluorination, highlighting gas dispersed CAP as a promising chemical-free and energy-efficient technology for PFAS remediation in water systems.

**Supplementary Information:**

The online version contains supplementary material available at 10.1038/s41598-026-57490-6.

## Introduction

**Fig. 1:**
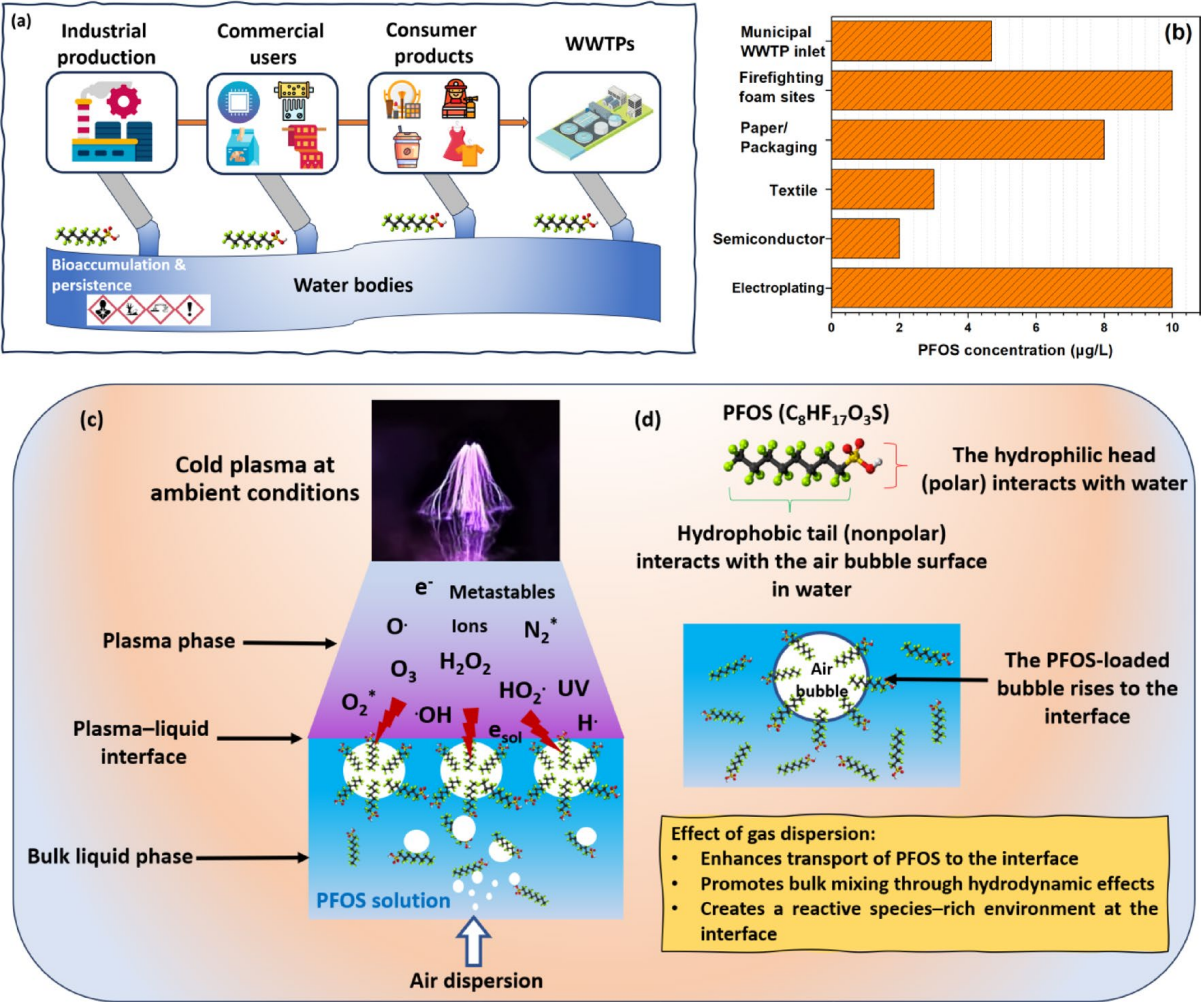
(**a**) Sources of PFAS in the environment. (**b**) A wide range of PFOS concentrations has been reported in various effluents, depending on the source, ranging from ng/L to µg/L and even mg/L^9,11–14^. (**c**) Schematic illustration of CAP-water interactions in the presence of air dispersion, showing the plasma-induced reactive species and reaction pathways proposed to contribute to PFOS degradation. (**d**) Mechanism of PFOS enrichment at air-bubble interface and transport to CAP-liquid interface.

Per- and polyfluoroalkyl substances (PFAS) are a large class of synthetic organofluorine compounds that have been widely used for decades. With over 15,000 individual chemicals identified, PFAS are defined by exceptionally strong carbon–fluorine (C–F) bonds, which confer remarkable chemical and thermal stability, as well as resistance to water and oil^1,2^. Their persistence is mainly attributed to the high bond dissociation energy of the C–F bond (~ 405–551 kJ/mol) and molecular structures that shield reactive carbon centers^3–5^. These unique physicochemical properties have led to the widespread applications of PFAS in firefighting foams, textiles, non-stick coatings, and numerous industrial processes (Fig. 1a)^6–8^. However, these same attributes also render PFAS highly persistent in the environment and in organisms, driving their designation as “forever chemicals.” PFAS are now widely detected in rivers, lakes, groundwater, and drinking water supplies, with around 23,000 contaminated sites identified across Europe^8–10^. Figure 1b summarizes representative PFOS concentrations (in µg/L) in water from various effluents, compiled from reported values, with concentrations ranging from ng/L to µg/L and even mg/L; the wide variability reflects differences in source characteristics, treatment processes, and site-specific conditions^9,11–14^.

Long-chain PFAS, such as PFOS and PFOA, can bioaccumulate in humans and wildlife and are linked to adverse health effects, including immunotoxicity, developmental toxicity, and increased cancer risk^5,15–17^. Their persistence and mobility have raised major environmental and public health concerns, leading to increasing regulatory restrictions. PFOS has been listed under the Stockholm Convention on Persistent Organic Pollutants and restricted under the European Union Persistent Organic Pollutants Regulation for more than a decade. Under new EU drinking water rules effective from January 2026, Member States are required to monitor PFAS systematically in drinking water and ensure that concentrations of the sum of 20 specific PFAS do not exceed 0.1 µg/L^18–20^. Germany is implementing these requirements through its updated Drinking Water Ordinance, including a sum PFAS-20 limit of 0.1 µg/L from January 2026 and an additional sum PFAS-4 limit of 0.02 µg/L for PFOS, PFOA, PFHxS, and PFNA from January 2028, based on health-based intake recommendations from the European Food Safety Authority. These stringent regulations and upcoming stricter guidelines on PFAS monitoring and allowable concentrations highlight the urgent need for effective PFAS degradation technologies beyond conventional treatment methods.

Conventional wastewater treatment plants (WWTPs) are largely ineffective at removing PFAS^5,21,22^. Adsorption-based technologies, including activated carbon, ion-exchange resins, and membrane filtration, can physically capture PFOS from aqueous media but do not achieve its chemical breakdown or defluorination^21,23–25^. In these processes, PFOS is transferred from the aqueous phase to a solid phase without alteration of its molecular structure, thereby generating secondary waste streams that require further treatment or disposal. Incineration is commonly used for PFAS destruction, typically requiring temperatures above 1000 °C; however, this process is highly energy-intensive, involves substantial operational and transportation costs associated with off-site waste handling, and may release hazardous by-products, raising economic and sustainability concerns^26,27^. Advanced reduction processes (ARPs) and advanced oxidation processes (AOPs) have been explored for PFOS degradation and defluorination^1,28–30^. Among these, ARPs based on hydrated (solvated) electrons (*e*⁻_sol_) show greater promise, as *e*⁻_sol_ are highly reactive reductive species that may contribute to electron transfer reactions with PFOS, thereby initiating hydrodefluorination and C–F bond cleavage. In contrast, AOPs generally achieve only partial degradation of PFOS, as this compound and PFAS more broadly lack C–H bonds and C–C π systems, making them highly resistant to attack by hydroxyl radicals (•OH), ozone (O₃), and other oxidizing species^30,31^.

CAP has recently emerged as a promising technology for the treatment of highly persistent emerging contaminants, including PFAS^31–33^. It simultaneously generates both oxidative and reductive reactive species at the gas–liquid interface, including *e*⁻_sol_, •OH, atomic hydrogen (H•), and a range of short- and long-lived plasma-derived species (as shown in Fig. 1c)^25,29,34–38^. Among these, *e*⁻_sol_ is of particular importance due to their exceptionally strong reducing power, with an extremely negative standard reduction potential (− 2.9 V) and lifetimes exceeding 300 μs, making them among the most powerful aqueous reductants^28,30,35,39^. *e*^−^_sol_ have been reported to react with PFAS, with rate constants on the order of ~ 10^6^–10^10^ M⁻^1^ s⁻^1^, and are proposed to contribute to C–F bond breakdown and subsequent defluorination^4,40^. In parallel, CAP also produces oxidative species, including •OH, O₃, singlet oxygen (O•), and other plasma-derived oxidants, which contribute to the degradation of reactive PFOS intermediates and TPs^31,36^. The synergistic action of reductive and oxidative species enhances both PFOS breakdown and defluorination. Importantly, CAP operates without chemical or catalyst additives and uses air as the working gas, making it an accessible, cost-effective, sustainable, and scalable approach for PFOS remediation.

CAP-liquid interactions in the presence of gas dispersion have emerged as an active area of research for the treatment of emerging contaminants and other applications, as they enable the generation of high concentrations of reactive species at phase boundaries^31,34,36,37,41–43^. Gas injection into liquids induces hydrodynamic effects and bubble formation that enhance plasma-liquid contact and facilitate the transport and accumulation of PFAS at the plasma-liquid interface^29,31,34,36^, as illustrated in Fig. 1d. This interfacial region is considered important for PFAS transformation and defluorination processes. In gas-dispersed CAP systems, PFOS molecules preferentially accumulate at the plasma-air–liquid interface, with the hydrophobic fluorinated chains oriented toward the gas phase and the hydrophilic head groups remaining in the aqueous phase. This interfacial arrangement may create a chemically active reaction zone where PFOS is exposed *e*⁻_sol_, short-lived reactive species, radicals, and other plasma-generated species. In addition, PFOS molecules at the interface may be exposed to gas-phase plasma species, including hot electrons (*e*_gas_), metastables, ions, and UV radiation, which can contribute to PFOS transformation and partial defluorination. Gas bubbling also promotes mixing and mass transport, enriches PFOS at interfacial and foam regions, and facilitates the transfer of reactive species into the bulk liquid.

Previous studies have demonstrated that synergistic interactions between plasma chemistry and bubble-induced dynamics can substantially enhance the generation of reactive species at interfacial regions and within the bulk liquid^29,31,36,39,44–46^. While CAP has shown strong potential for PFAS degradation, significant challenges remain, including inefficient interactions between PFAS and reactive species and the low utilization of short-lived species that are critical for rapid degradation and defluorination. Bubble-mediated processes offer a promising pathway to address these limitations by increasing interfacial area and facilitating the transport of surface-active compounds such as PFOS toward the plasma-liquid interface, where short-lived reactive species (e.g. *e*⁻_sol_ and •OH) can react most effectively. However, the mechanistic role of gas-induced hydrodynamics in CAP systems remains an active field of research. In particular, the combined effects of gas dispersion, interfacial enrichment, and plasma chemistry on degradation kinetics and defluorination efficiency have received limited systematic investigation. However, these effects have recently become a major focus of research due to their critical importance for scalable applications and for the effective treatment of a wide range of PFAS, including both long- and short-chain compounds. Furthermore, most existing studies provide limited investigation of fluorine mass balance and offer little insight into transformation pathways, particularly for persistent short-chain PFAS, their degradation, and the underlying degradation mechanisms.

In this study, we address these gaps by introducing a novel CAP reactor integrating controlled gas dispersion to enhance interfacial reactions and maximize the utilization of short-lived reactive species. The system promotes dynamic enrichment of PFOS and its TPs, including short-chain PFAS, at continuously renewed interfaces, thereby improving contact with reactive species and enabling accelerated degradation and partial defluorination. We systematically investigate PFOS degradation in tap water under ambient conditions, with a particular focus on the role of gas dispersion in governing mass transport and reaction efficiency. This study provides a comprehensive fluorine mass balance, detailed kinetic analysis, and new insight into degradation pathways. Our results highlight the importance of gas-induced hydrodynamic effects in enhancing plasma-liquid interactions and demonstrate the potential of gas-dispersed CAP as a scalable, energy-efficient, and rapid strategy for PFAS remediation.

## Materials and methods

### PFOS solution for treatment

A high-concentration PFOS solution was prepared in a tap water matrix to enable the systematic investigation of its degradation and partial defluorination behavior under realistic aqueous conditions. The use of tap water was intended to better simulate environmentally relevant water chemistry. A stock solution of PFOS (Sigma-Aldrich, ≥ 98% purity) was initially prepared at 200 mg/L in tap water and subsequently diluted to obtain a working concentration of 5 mg/L. The high initial concentration was selected to minimize experimental errors due to adsorption losses and to allow for accurate measurement of PFOS degradation over time. Although PFOS concentrations in environmental water sources depend on the sources and are typically in the ng/L to mg/L range, reported levels span several orders of magnitude. Distinct differences in concentration ranges were observed between sites located within or near the sources. In this study, the elevated concentration ensures reliable analytical detection and allows for accurate kinetic analysis.

The initial PFOS solution also contained a low concentration of perfluorohexanesulfonate (PFHxS = 3.96 µg/L at an initial PFOS concentration of approximately 5 mg/L), likely originating as an impurity in the PFOS standard solution. Accordingly, PFHxS was also monitored during CAP treatment. However, because the initial PFHxS concentration was several orders of magnitude lower than that of PFOS, its degradation behavior and kinetics are not directly comparable to those of PFOS. In addition, differences in concentration-dependent plasma-liquid interfacial interactions, transport behavior, surface activity, and analytical sensitivity may further influence the observed PFHxS degradation trends under CAP treatment conditions.

### CAP with air dispersion experimental setup

**Fig. 2:**
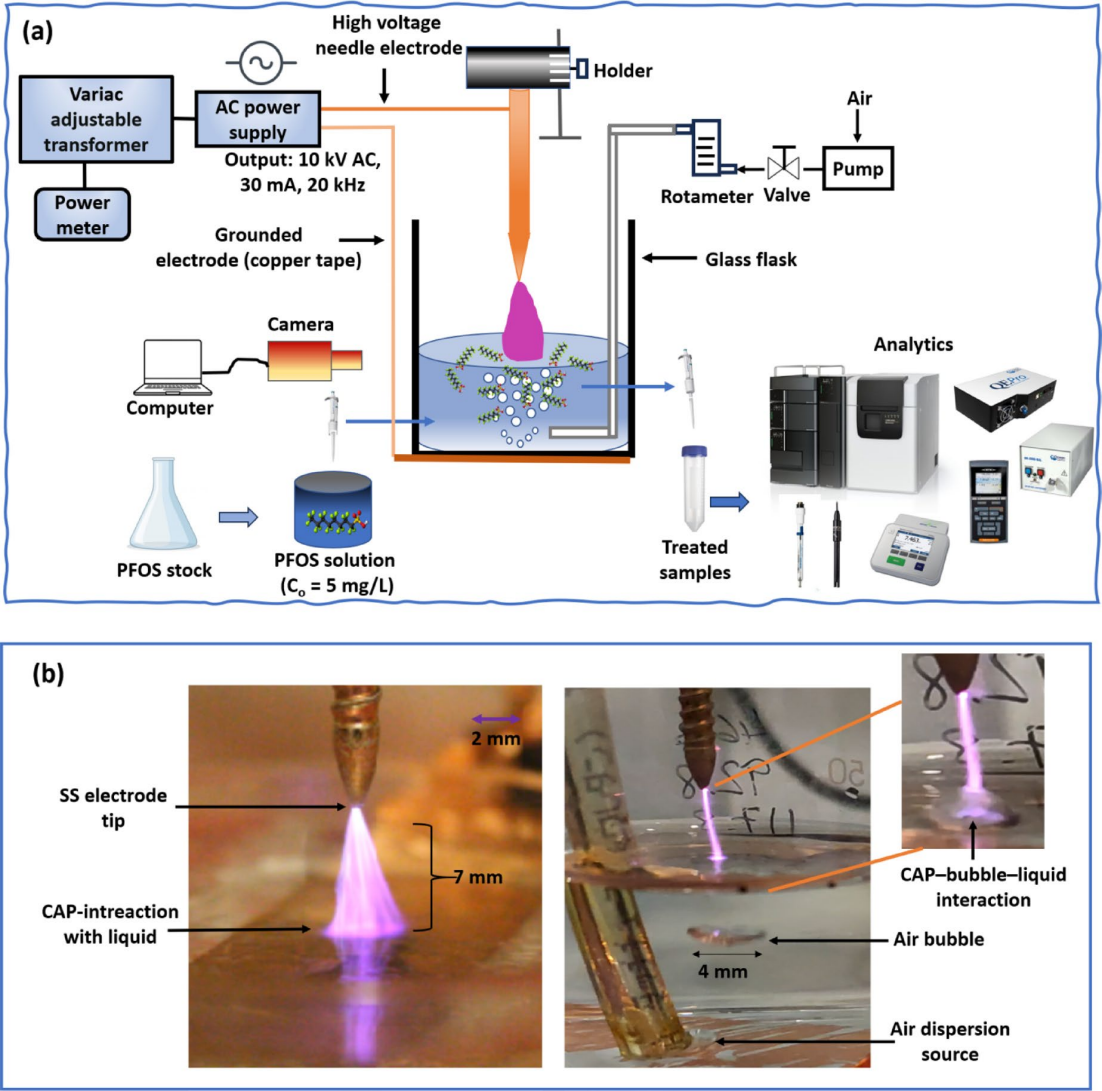
(**a**) CAP system with gas-dispersion configuration for PFOS treatment and measurement of reactive species. (**b**) CAP interactions with the liquid phase under two conditions: stationary liquid (left) and with air dispersion (right), illustrating plasma-bubble–liquid interactions.

CAP was generated in ambient air above the liquid surface, with and without air dispersion into the liquid. A schematic of the experimental setup is shown in Fig. 2a. The CAP was produced using a stainless-steel needle electrode (2 mm diameter with a 1 mm tip) positioned approximately 7 mm above the liquid surface in a glass Petri dish containing 10 mL to 40 mL of PFOS solution. The sharp-tipped needle was selected to generate intense local electric fields, facilitating plasma ignition and promoting the formation of high concentrations of reactive species^47,48^. The CAP images with and without gas dispersion (air bubble generation in the liquid phase) were captured using a digital single-lens reflex (DSLR) camera (shown in Fig. 2b).

The CAP was powered by a high-voltage AC power supply (NeonPro NP-10000–30; 10 kV, 30 mA) with a variable transformer (Hossoni SV2-A, 500 VA) to adjust output voltage. One terminal was connected to the needle electrode, and the other to copper tape wrapped around the bottom of the Petri dish. Input power was monitored using a digital power analyzer, showing minor fluctuations over time due to changes in plasma treated liquid characteristics. The average power delivered for plasma generation was about 12 W, which enabled stable plasma ignition at reduced input voltage and minimized fluctuations.

For experiments with air dispersion, air bubbles were introduced through a silicone tube (inner diameter 4 mm, outer diameter 6 mm) connected to an ambient air pump. The power required to generate continuous bubbles in the liquid by using the pump was much lower than that required to sustain the CAP. The airflow rate was maintained at 0.1 L/min using a rotameter, generating bubbles at the bottom of the Petri dish that rose to the liquid surface at the CAP-liquid interaction region. Owing to the surfactant nature of PFOS, the molecules preferentially accumulated at the gas–liquid interface, leading to the formation of an ultrathin PFOS-enriched interfacial layer and a faint foam film near the liquid surface. This interfacial enrichment increased the local PFOS concentration at the plasma-liquid boundary, thereby enhancing exposure to short-lived reactive species generated by CAP. The formation, stability, and thickness of this PFOS-rich interfacial layer, as well as possible PFOS disappearance through adsorption onto reactor walls/electrodes or partitioning into foam layers under both plasma and non-plasma gas-dispersion conditions, require further investigation. Future studies will therefore employ optical imaging, surface-tension analysis, and other interfacial characterization techniques to better understand PFOS transport and interfacial behavior during plasma treatment.

In addition, air dispersion induced bulk mixing within the solution, facilitating the transport of PFOS and its degradation products, including short-chain PFAS, toward the plasma-liquid interface. This configuration enabled investigation of the synergistic interactions within the plasma-air dispersion-liquid three-phase system and their influence on PFOS degradation, transformation, and defluorination. All experiments were conducted in a closed system, and an exhaust line was installed above the reactor to prevent possible accumulation of PFAS in the gas phase during operation. The CAP-liquid interaction under gas dispersion is shown in Supplementary Video 1, where the presence of air bubbles enhances PFOS enrichment at the gas–liquid interface, intensifies plasma-liquid contact, and promotes the formation of interfacial microdischarges at the bubble–liquid boundary, thereby enhancing the generation and transport of reactive chemical species. For experiments without gas dispersion, CAP interacted with a stationary liquid surface. All the experiments were conducted at room temperature (22 ± 2 °C).

### Analysis of physicochemical properties of CAP-treated sample

The physicochemical properties and reactive species formation in CAP-treated liquids were characterized using a range of analytical tools and chemical probes. Both tap water (without PFOS) and PFOS-containing water were treated under varying CAP exposure times, with and without air dispersion. Tap water, which does not contain PFOS, was used to evaluate reactive species generation in the absence of PFAS consumption. The physicochemical properties of both systems were measured to provide insight into reactive species formation during CAP treatment and their interactions with the liquid phase.

The pH (METTLER TOLEDO™ InLab™ Routine pH electrode), conductivity (WTW™ IDS Conductivity Cell TetraCon™ 925/LV with MultiLine® 3620 IDS device), and oxidation–reduction potential (ORP, xylem, electrode SensoLyt® ORP 900-P) were measured while liquid temperature was monitored with a Mesee LCD Digital Water Quality Tester. Hydrogen peroxide (H_2_O_2_) concentrations were determined using ITS Europe WaterWorks Peroxid H₂O₂ Test Strips (480,014), and the corresponding •OH concentration was estimated, considering that H₂O₂ predominantly forms via recombination of two •OH, providing insight into reactive species generated at the gas phase, plasma-liquid interface, and within the bulk liquid. UV–Vis absorption spectra of plasma treated water were recorded from 210 to 250 nm using an Ocean Optics QEPro spectrometer coupled with a DH-2000-BAL light source and QP600–2-UV/VIS optical fiber to qualitatively identify plasma-induced reactive species.

The steady-state concentration of *e*⁻_sol_ was estimated indirectly from PFOS degradation kinetics, based on their high reported reactivity with PFOS (rate constant ~ 1 × 10⁹ M⁻^1^ s⁻^1^). Direct measurement of *e*⁻_sol_ in plasma–liquid systems remain extremely challenging due to their ultrashort lifetime and limited penetration depth in water during CAP-liquid interactions. Although some studies have attempted to probe *e*⁻_sol_ in plasma-treated liquids^35,49^, quantitative determination under dynamic, gas-dispersed conditions remains an active area of research. In the presence of gas dispersion, PFOS decay followed apparent first-order kinetics. Assuming that PFOS degradation was dominated by reactions with *e*⁻_sol_, under conditions of effective mixing and minimal scavenging by competing species, the calculated *e*⁻_sol_ concentrations represent conservative lower-bound estimates of the electron densities required to achieve the observed PFOS degradation. The calculation procedure for *e*⁻_sol_ concentration is provided in the Supplementary Information.

### Measurement of PFOS concentrations by LC–MS

The concentrations of PFOS, PFHxS, and their degradation products, including the perfluorocarboxylate intermediates PFBA, PFPeA, PFHxA, PFHpA, and PFOA, were quantified using liquid chromatography-mass spectrometry (LC–MS; Shimadzu, Jena, Germany) equipped with a Gemini C6-Phenyl column (100 mm length, 2 mm ID; Phenomenex, Aschaffenburg, Germany) with a particle size of 3 µm and a pore size of 110 Å, maintained at a column temperature of 40 °C. Chemical structures were generated using the open-source MolView application (shown in Table 1). The mobile phase consisted of solvent A (20 mM ammonium acetate in 10 vol % methanol and 90 vol % deionized water) and solvent B (20 mM ammonium acetate in 10 vol % deionized water and 90 vol % methanol), with a gradient of 65 vol % B and 35 vol % A delivered at 0.25 mL/min over a 28 min run. Detection limits were 0.6 µg/L for PFOS, 0.2 µg/L for PFHxS, and 0.2–2 µg/L for other PFAS intermediates, with a standard deviation within 2% of the mean. After the treatment, the samples were collected in vials and transported to another laboratory for HPLC–MS analysis and fluoride ion measurements.

**Table 1:**
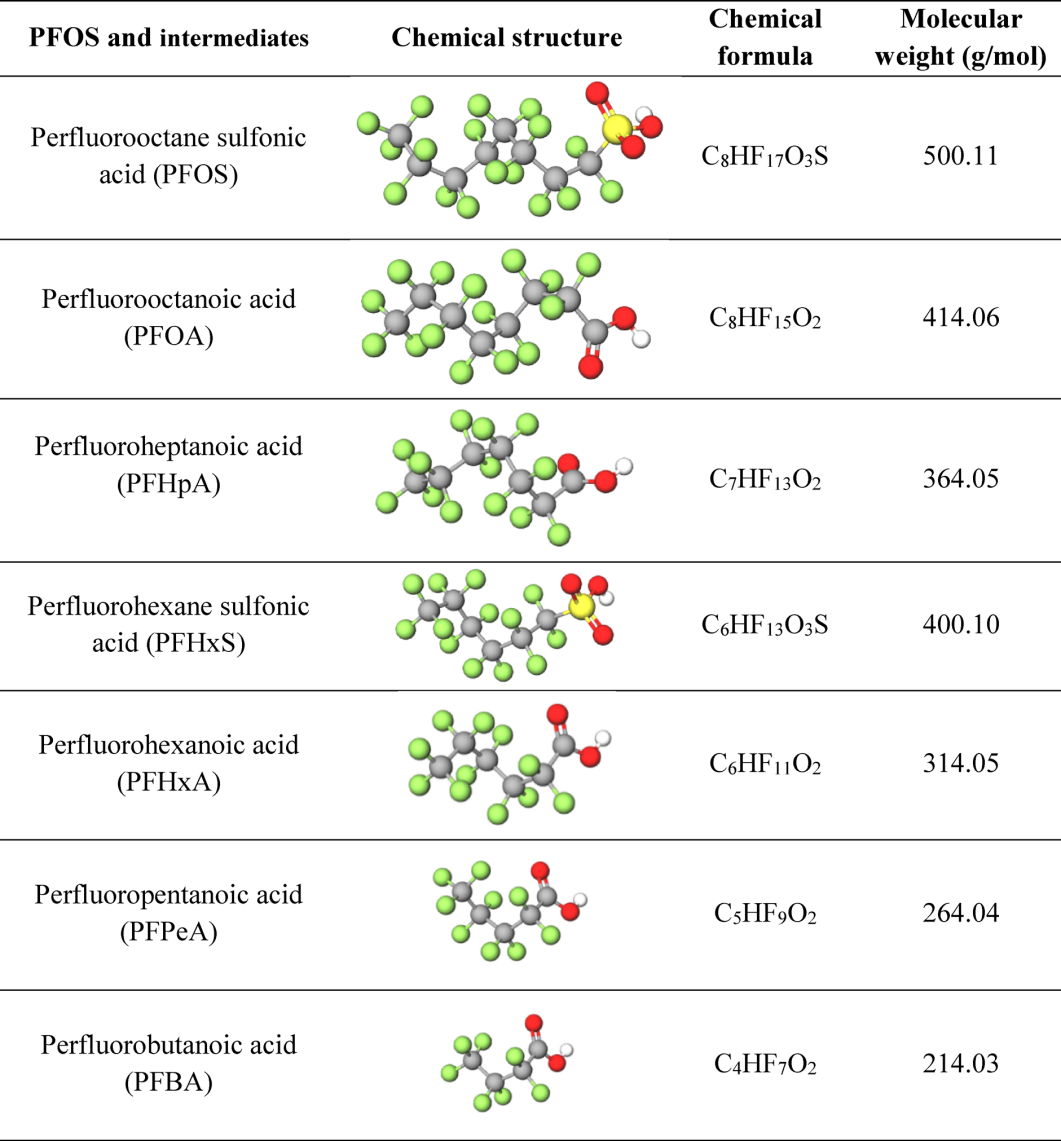
Detailed chemical structures, formulas, and molecular weights of PFOS, PFHxS, and the measured TPs.

Released fluoride ions during PFOS degradation were measured using the Spectroquant® Fluoride test reagent (Supelco, 1.17236.0001) based on the SPADNS method, analogous to USEPA Method 340.1, in which fluoride reacts with an arsenic-free red zirconium-dye solution to form a colorless complex, and the unconsumed dye is quantified photometrically at 575 nm. Samples containing 0.1–2 mg/L fluoride were mixed with the reagent in a 10:1 volume ratio, and absorbance was measured after 20 min. This combined analytical approach enabled simultaneous quantification of PFOS, its degradation intermediates, and released fluoride, providing comprehensive insights into the partial defluorination kinetics and transformation pathways.

The mean values and standard deviations of the HPLC–MS measurements are provided in Supplementary Table S1. The small standard deviations indicate that the measured values were closely clustered around the mean. Accordingly, the error bars in the PFOS concentration–time profiles (Supplementary Fig. S1) are minimal, demonstrating the high reproducibility and precision of the analytical measurements. All calculations used in this study are detailed in the Supplementary Information (Text S1).

## Results and discussion

### Physicochemical investigation of CAP-treated water

**Fig. 3:**
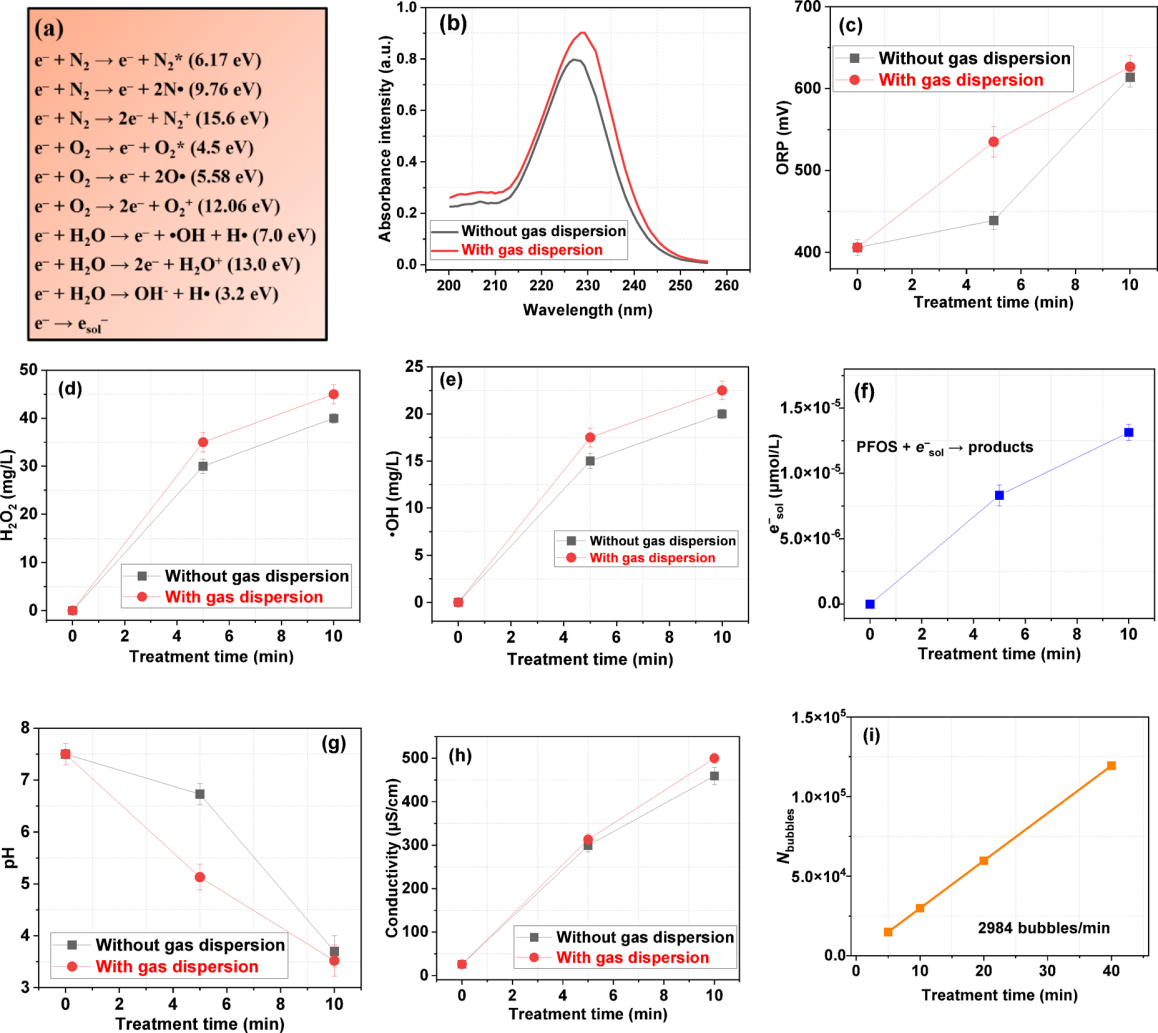
CAP interaction with liquid and generation of physicochemical effects. (**a**) Possible electron-impact and plasma-liquid reactions. (**b**) Optical absorbance spectra (after 5 min of treatment). (**c**) Variation of ORP with treatment time. (**d**) H_2_O_2_ concentration with treatment time. (**e**) •OH concentration with treatment time (calculated from measured H_2_O_2_ concentration). (**f**) Solvated electron concentration over time (with gas dispersion), measured indirectly based on PFOS degradation kinetics. (**g**) Change in pH with treatment time. (**h**) Variation in water conductivity with treatment time. (**i**) Number of bubbles generated with treatment time. Experiments were conducted at a gas-dispersion flow rate of 0.1 L/min, input power of 12 W, and V_0_ of 10 mL.

CAP generated in ambient air above the liquid surface creates a highly reactive nonequilibrium environment containing energetic electrons (typically up to ~ 10 eV), ions, UV radiation, and reactive oxygen and nitrogen species. Although the bulk liquid remains near ambient temperature, electron-impact processes efficiently ionize and excite N_2_, O_2_, and H_2_O, thereby generating reactive species that contribute to the degradation of highly persistent contaminants such as PFOS. In the gas phase, electron collisions produce excited and ionized species, atomic oxygen and secondary electrons, which collectively sustain coupled gas- and liquid-phase reaction pathways (as some of possible reactions are shown in Fig. 3a)^33,41,50,51^. At the plasma-liquid interface, a fraction of low-energy plasma electrons penetrates into the aqueous phase, where they undergo rapid energy loss through frequent elastic and inelastic scattering events. Upon thermalization, these electrons become localized within transient solvent cavities and are stabilized as *e*⁻ₛₒₗ. CAP thus establishes a spatially confined interfacial reactive zone enriched in *e*⁻ₛₒₗ and other short-lived species^35,40,52,53^. Owing to their ultrashort lifetimes and limited diffusion lengths, *e*⁻ₛₒₗ are expected to be concentrated near the interface, where they may react with PFOS and its TPs, and are proposed to contribute to C–F bond cleavage and defluorination^4,30,37,38,40^.

CAP, applied with and without gas dispersion into the liquid, was used to investigate the physicochemical properties of plasma-treated water and to elucidate the formation of long-lived reactive species. The generation of these species was monitored using time-resolved UV–Vis absorption spectroscopy, ORP measurements and other analytical probes. UV–Vis absorption spectra recorded in the 210–250 nm range under both gas-dispersed and non-dispersed conditions (shown in Fig. 3b) exhibit a characteristic absorption band associated with long-lived reactive oxygen and nitrogen species, including H₂O₂ and oxidized nitrogen–oxygen products^54,55^. Gas-dispersed CAP produced higher absorbance, indicating enhanced accumulation of long-lived reactive species in water under bubble-assisted conditions, primarily due to intensified mass transfer and enhanced interfacial interactions at the gas–liquid plasma boundary. The oxidative strength of plasma-treated water was further assessed through time-dependent ORP measurements (shown in Fig. 3c). ORP is a key indicator of the oxidizing-reducing conditions of an aqueous system^56,57^. The ORP increased progressively with plasma exposure time, confirming enrichment of the liquid phase with oxidizing species. This increase was substantially more pronounced under gas-dispersed conditions, reaching 535 mV after 5 min and 626 mV after 10 min, compared with non-dispersed treatment.

H_2_O_2_, a representative long-lived reactive oxygen species in water, was quantified. As shown in Fig. 3d, H_2_O_2_ concentration increased nearly linearly with plasma treatment time. Notably, significantly higher concentrations were observed in the presence of gas dispersion, reaching 35 mg/L after 5 min and 45 mg/L after 10 min. This enhancement in the concentration is attributed to more efficient generation and transfer of reactive species at the gas–liquid interface, as well as intensified recombination of •OH within the bulk liquid. H_2_O_2_ can be produced via gas-phase radical recombination followed by dissolution into the liquid, as well as through •OH recombination occurring at the plasma-liquid interface and within the bulk solution^47,56,58^. The accumulation of H_2_O_2_ in the bulk liquid therefore provides indirect but compelling evidence for •OH generation during CAP-liquid interactions. Consistent with this interpretation, the effective concentration of •OH in the bulk liquid, inferred from H_2_O_2_ measurements, increased with plasma treatment time (Fig. 3e). Gas injection resulted in higher steady-state •OH concentrations, suggesting that the enlarged gas–liquid interfacial area and locally enhanced electric fields at bubble surfaces promote •OH generation. The presence of dispersed gas bubbles introduces regions of lower electrical conductivity and dielectric discontinuity within the liquid phase, which can concentrate the electric field at the gas–liquid interface^29,59^. In addition, the curved bubble surface may further amplify the local field intensity, facilitating electron-driven dissociation of water molecules and subsequent •OH formation.

It is important to note that H_2_O_2_-based estimation provides only an indirect measure of •OH activity. It cannot distinguish between interfacial and bulk •OH formation or capture the short-lived, spatially localized nature of •OH. In addition, although H_2_O_2_ formation is primarily governed by •OH recombination, other plasma-induced pathways and secondary reactions may also contribute. In our previous studies, selective chemical probe compounds such as succinic acid and p-chlorobenzoic acid were used to quantify •OH formation in the liquid phase based on probe oxidation reactions^47,60^. The formation of •OH in the gas phase under CAP conditions was confirmed in our previous work using optical emission spectroscopy (OES) at 309 nm, supported by intensified charge-coupled device (ICCD) imaging with a band-pass filter to visualize the spatial emission profile of •OH^60,61^. These measurements also provide evidence for the transport of •OH radicals toward the plasma-liquid interface and their subsequent transfer into the bulk liquid phase.

The generation of *e*⁻ₛₒₗ by CAP was indirectly estimated from the kinetics of PFOS degradation, assuming first-order behavior for the reaction between PFOS and *e*⁻ₛₒₗ. The use of PFOS as an indirect probe for *e*⁻ₛₒₗ is supported by its strong electron-scavenging capacity and reported reactivity toward solvated electrons. Under these assumptions, reactions involving *e*⁻ₛₒₗ are proposed to contribute to the observed PFOS degradation. As shown in Fig. 3f, the estimated steady-state concentration of *e*⁻ₛₒₗ (µmol/L) increased with CAP treatment time under gas-dispersed conditions, based on the experimentally observed first-order PFOS decay. Although *e*⁻ₛₒₗ was not directly measured in this study, the results are consistent with sustained electron injection into the liquid phase and suggest that reductive pathways may contribute to PFOS transformation. The inferred steady-state *e*⁻ₛₒₗ concentration reflects a dynamic balance among electron generation in the plasma phase, interfacial transport across the plasma-liquid boundary, and consumption by PFOS and other reactive species in solution. While *e*⁻ₛₒₗ have been proposed in previous studies to contribute to PFOS defluorination through reductive pathways, their specific role in the present CAP system cannot be directly confirmed. In addition to *e*⁻ₛₒₗ, other plasma-generated reactive species, including •OH, reactive oxygen and nitrogen species, ions, metastables, and UV-induced processes, may also contribute to PFOS transformation and defluorination. Therefore, indirect estimation based solely on PFOS decay cannot fully distinguish the relative contributions of individual reactive species. This limitation is inherent to indirect reactive-species analysis in complex plasma-liquid systems, where multiple oxidative and reductive pathways may occur simultaneously and exhibit overlapping reaction kinetics.

Direct detection of *e*⁻ₛₒₗ at plasma-liquid interfaces remains experimentally challenging due to their ultrashort lifetimes and spatial confinement near the interface. The estimated concentrations of *e*⁻ₛₒₗ in this work are to some extent supported by previous reports. For example, Rumbach et al.^35^ directly detected plasma-generated *e*⁻ₛₒₗ near aqueous interfaces using diode-laser-modulated absorption spectroscopy, reporting low micromolar concentrations confined to nanometre-scale interfacial regions. Other studies have also discussed the formation and characterization of *e*⁻ₛₒₗ in plasma-liquid systems^49,62,63^. Further investigations are needed for the direct detection and quantification of *e*⁻ₛₒₗ in gas-dispersed CAP systems, given their high reactivity, extremely short lifetimes, and confinement to interfacial regions.

The evolution of pH and electrical conductivity was monitored to evaluate the transport and accumulation of plasma-generated reactive species in the liquid phase during CAP treatment, both with and without gas dispersion. As shown in Fig. 3g, the pH of the treated water decreases monotonically with treatment time, with a more pronounced effect observed under gas-dispersed CAP conditions. The observed pH decrease is primarily attributed to the generation and dissolution of long-lived nitrogen species in the liquid^47,61^. Consistent with these observations, the electrical conductivity of the solution increases steadily over time (shown in Fig. 3h). Higher conductivity values are observed in the presence of gas dispersion, indicating greater accumulation of ionic species^47^. These physicochemical changes induced by CAP are expected to directly influence PFOS degradation and defluorination behavior.

The number of bubbles generated per minute during gas dispersion, calculated based on the flow rate of air introduced into the water, grows nearly linearly with treatment time and reaches about 3000 bubbles/min at the longest treatment, as shown in Fig. 3i. This confirms the stable formation of bubbles and underscores their role in providing a large interfacial area, which enhances mass transfer and strengthens the overall plasma-liquid coupling.

### PFOS degradation and partial defluorination by CAP treatment

**Fig. 4:**
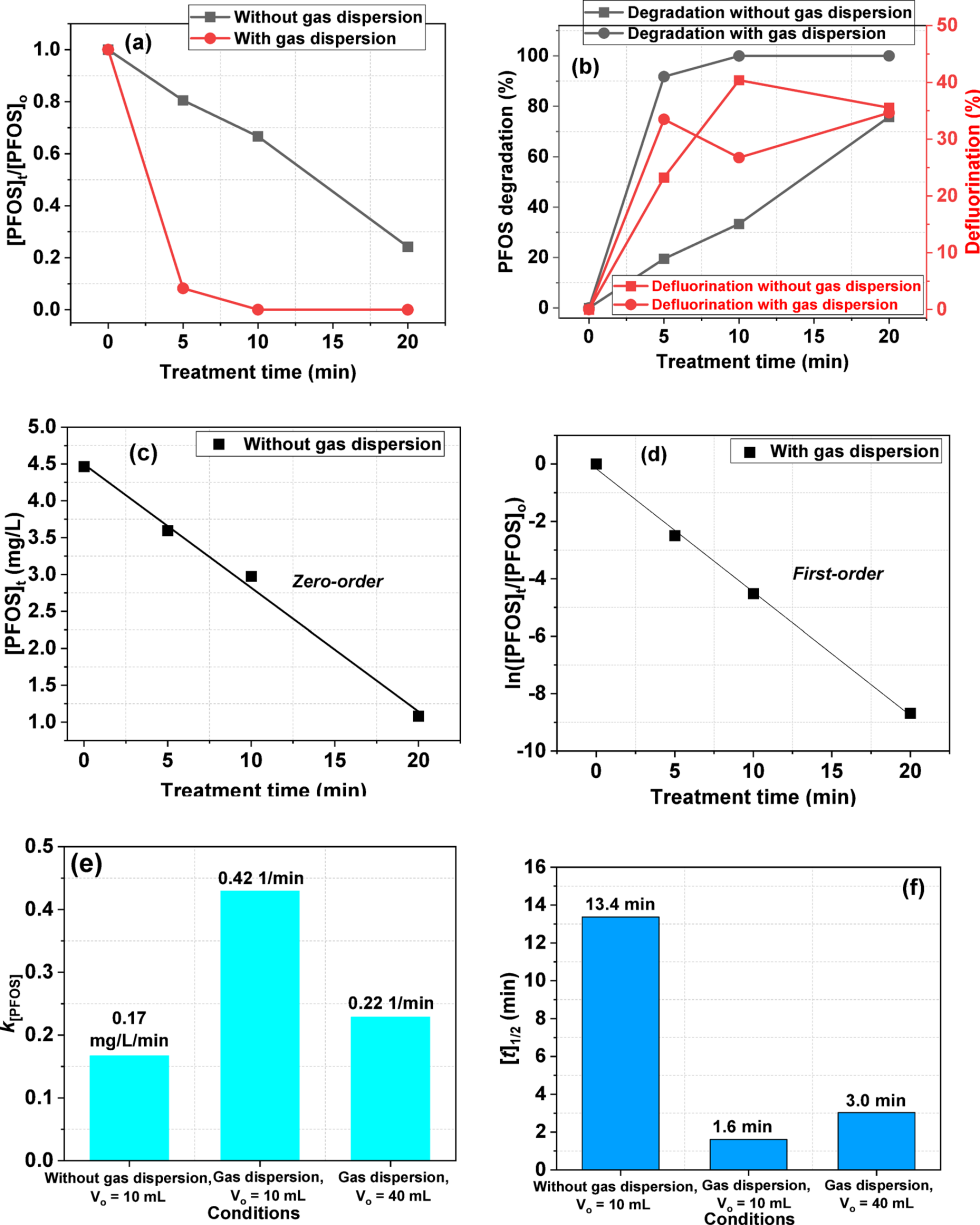
PFOS degradation, partial defluorination, and kinetics with and without gas dispersion. (**a**) Shows the reduction in PFOS concentration over treatment time (V₀ = 10 mL). (**b**) The degradation and partial defluorination of PFOS over the same treatment period (V₀ = 10 mL). (**c**) Zero-order kinetics without gas dispersion (V₀ = 10 mL). (**d**) First-order kinetics with gas dispersion (V₀ = 10 mL). (**e**) The comparison of PFOS degradation rates under experimental conditions using two different initial volumes. (**f**) The half-life of PFOS degradation. The experiments were conducted with a gas-dispersion flow rate of 0.1 L/min and input power of 12 W.

This section discusses PFOS reduction, degradation, and partial defluorination during CAP treatment, both with and without gas dispersion. As shown in Fig. 4a, the normalized PFOS concentration decreases monotonically with treatment time under all conditions. Notably, the rate of PFOS reduction is markedly higher in the presence of gas dispersion than in its absence. Figure 4b presents the evolution of PFOS degradation and partial defluorination efficiencies. Quantitatively, without gas dispersion, PFOS degradation reaches only 19% after 5 min and increases to 76% after 20 min of treatment. In contrast, the application of gas dispersion dramatically accelerates PFOS degradation, achieving 92% degradation within the first 5 min, 99% after 10 min, and 99.99% within 20 min. These results highlight the critical role of bubbles in promoting interfacial reactions and enhancing the effective utilization of plasma-generated reactive species.

The extent of defluorination consistently lags behind PFOS degradation, particularly at shorter treatment times, and is lower than the overall degradation efficiency. This indicates that PFOS transformation likely proceeds through the formation of partially defluorinated intermediates, while C–F bond cleavage and fluoride release occur progressively during extended treatment. In the absence of gas dispersion, the defluorination exhibits a fluctuating trend, increasing from 23% at 5 min to 40% at 10 min, followed by a decrease to 36% at 20 min. A similar non-monotonic behavior is observed under gas-dispersed conditions, where defluorination reaches 33% after 5 min, decreases to 27% at 10 min, and then increases again to 35% after 20 min. These fluctuations suggest a dynamic balance between PFOS breakdown, intermediate formation, and fluoride release.

Several factors may contribute to the observed deviations in apparent defluorination efficiency. Fluoride released during PFOS degradation may undergo partial adsorption onto reactor surfaces or deposited reaction products, thereby reducing the measurable dissolved fluoride concentration. In addition, volatile or semi-volatile fluorinated intermediates generated during plasma treatment may partition into gas, foam, or condensed phases and therefore remain unquantified in the aqueous phase. Transient accumulation and subsequent degradation of fluorinated intermediates may further contribute to fluctuations in measured fluoride concentrations. Analytical variability associated with low-concentration fluoride measurements and the complex plasma-treated matrix may also influence the observed trends. Similar behavior has been reported in previous plasma-based PFAS treatment studies. For example, a related study by Lewis et al.^37^ reported high PFAS degradation but lower defluorination efficiencies during gliding arc plasma treatment, together with incomplete fluorine mass balance and intermediate formation. These observations support the interpretation that fluorine redistribution occurs during plasma treatment and highlight the need for integrated gas, liquid, and interfacial-phase fluorine analysis.

The influence of initial liquid volume was also investigated. Increasing the initial sample volume from 10 to 40 mL required longer treatment times to achieve high levels of PFOS degradation. As shown in Fig. S2 (Supplementary Material), under gas-dispersed CAP conditions, rapid PFOS degradation was observed even at the larger volume, reaching 77% within the first 5 min and nearly 99.9% degradation after 30 min of treatment. In contrast, PFOS defluorination proceeded more gradually, increasing from 17% at 5 min to 27% after 30 min. The reduced treatment efficiency at larger volumes is attributed primarily to transport and geometric limitations. At lower liquid heights, a larger fraction of the solution is directly exposed to the plasma-liquid interface, where PFOS accumulates and reacts rapidly. In contrast, larger volumes place more of the liquid beyond the reactive interfacial zone, making degradation increasingly dependent on diffusion and mixing. As a result, PFOS experiences a shorter effective residence time within the plasma-affected region. Because plasma operating conditions and chemistry were identical across all volumes, the observed differences in degradation and partial defluorination are attributed to transport and exposure constraints rather than changes in intrinsic plasma reactivity.

A kinetic analysis was conducted to elucidate the degradation order of PFOS under CAP treatment with and without gas dispersion. As shown in Fig. 4c, d and Fig. S3, PFOS degradation follows zero-order kinetics in the absence of gas dispersion, whereas the introduction of gas dispersion shifts the degradation behavior to first-order kinetics. Without gas dispersion, PFOS degradation is well described by a zero-order model, with a rate constant of 0.17 mg/L/min (R^2^ = 0.99). This kinetic behavior arises because the CAP is generated above a static liquid surface, creating a single, fixed plasma-liquid interface where plasma chemistry and reactive-species generation remain relatively constant during treatment. Due to its surfactant nature, PFOS preferentially accumulates at this interface, where degradation occurs within a thin reactive layer. Under these conditions, the degradation rate is governed primarily by the nearly constant flux of plasma-generated reactive species reaching the interface, rather than by the bulk PFOS concentration, resulting in concentration-independent (zero-order) kinetics. In addition, limited interfacial area and restricted mass transfer from the bulk liquid to the reactive zone further contribute to the observed zero-order behavior. In contrast, the introduction of gas dispersion fundamentally alters the plasma-liquid interaction regime.

As shown in Fig. 4e, PFOS degradation under gas-dispersed conditions follows first-order kinetics, with a rate constant of 0.42 1/min at a lower initial volume (V₀ = 10 mL; R^2^ = 0.99). Increasing the initial volume to 40 mL decreases the apparent rate constant to 0.22 1/min (R^2^ = 0.98), likely due to dilution effects and increased mass-transfer limitations of plasma-generated reactive species at greater liquid heights. Gas dispersion creates a dense population of transient bubble interfaces, substantially increasing the effective gas–liquid interfacial area. PFOS preferentially partitions to these interfaces, where it is exposed to plasma-generated reactive species, while enhanced mixing facilitates continuous transport from the bulk solution to the reactive zone. Under these conditions, degradation follows apparent first-order kinetics, reflecting reaction at dynamically renewed interfacial sites. Previous studies have likewise reported first-order PFOS degradation during cold plasma treatment under conditions involving bulk mixing. For example, Palma et al.^64^ reported rate constants of 0.107 1/min in ultrapure water and 0.0633 1/min in groundwater, while Alam et al.^34^ observed rate constants between 0.01 and 0.11 1/min under varying conditions. The higher reaction rate constants obtained in the present work suggest that gas dispersion enhances interfacial reactivity and mass transfer, thereby accelerating PFOS degradation and partial defluorination.

Figure 4f summarizes the PFOS half-lives obtained from kinetic analysis under different treatment conditions. The half-life, defined as the time required for 50% PFOS degradation, is substantially reduced under gas-dispersed CAP conditions and at lower sample volumes. Notably, gas dispersion yields shorter PFOS half-lives even at higher initial volumes compared to non-dispersed treatment at lower volumes. This pronounced reduction in half-life showed the strong synergistic effect of bubble-mediated plasma-liquid coupling and optimized hydrodynamics in accelerating PFOS destruction.

**Fig. 5:**
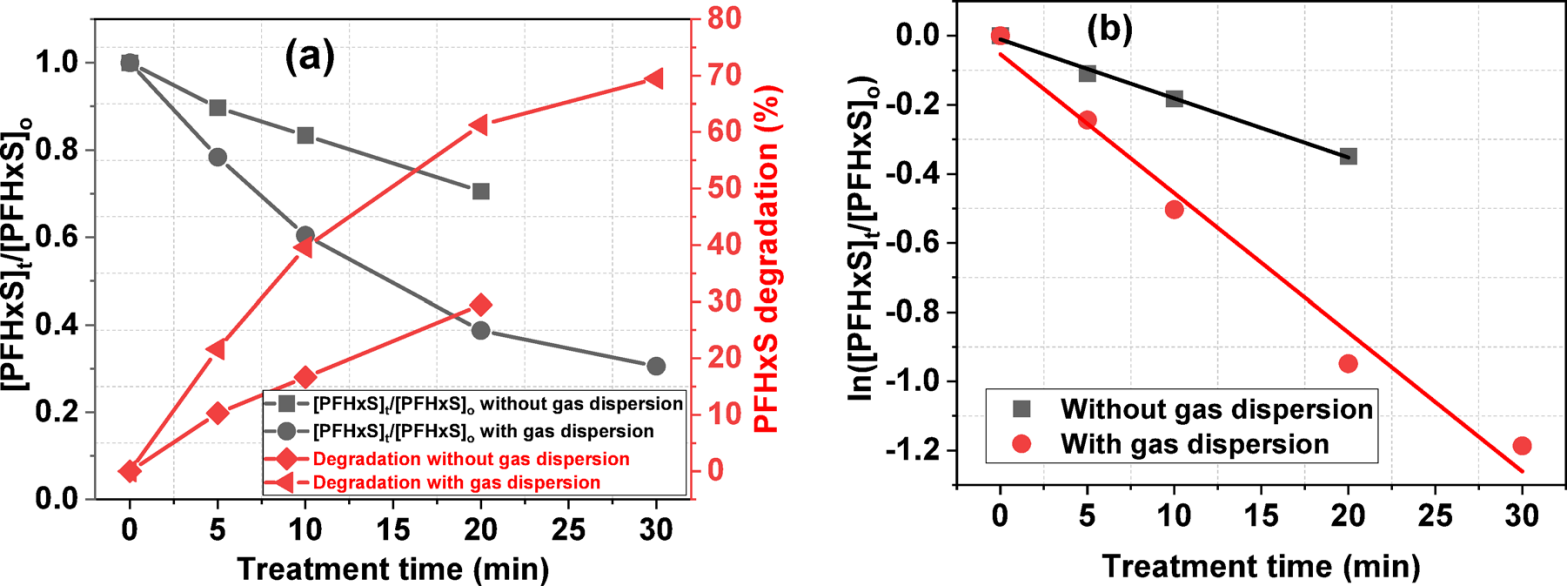
(**a**) The normalised reduction in PFHxS concentration and its degradation over time (V₀ = 10 mL). (**b**) The degradation kinetics of PFHxS (V₀ = 10 mL). The experiments were conducted with a gas-dispersion flow rate of 0.1 L/min and input power of 12 W.

The degradation behavior of PFHxS, which was present in the initial samples at concentrations several orders of magnitude lower than that of PFOS, was investigated as a function of CAP treatment time. As shown in Fig. 5a, the normalized PFHxS concentration decreases steadily with increasing treatment time under all conditions. The presence of gas dispersion further enhances PFHxS degradation, resulting in higher degradation efficiencies over the same exposure period. Specifically, after 20 min of treatment, PFHxS degradation reaches 29% without gas dispersion, compared to 61% with gas dispersion. Extending the gas-dispersed treatment to 30 min further increases PFHxS degradation to 69%.

The divergence between the concentration profiles obtained with and without gas dispersion indicates that the beneficial effect of bubble-assisted CAP is not limited to PFOS but also extends to shorter-chain perfluoroalkyl sulfonates such as PFHxS. However, the degradation behavior and kinetics of PFHxS are not directly comparable to those of PFOS due to inherent differences in molecular structure and interfacial activity. PFHxS typically exhibits weaker surfactant properties than PFOS, resulting in reduced interfacial enrichment and a shorter residence time within the highly reactive plasma-liquid interfacial region. In addition, its lower initial concentration further limits the probability of interaction with short-lived reactive species, contributing to lower apparent degradation rates. The introduction of gas dispersion partially overcomes these limitations by enhancing hydrodynamic mixing, increasing the gas–liquid interfacial area, and continuously transporting PFHxS from the bulk solution to the plasma-liquid interface. This improves the effective exposure of PFHxS to plasma-generated reactive species, including radicals, solvated electrons, and interfacial oxidative species, thereby promoting its degradation. Kinetic analysis further supports these observations, confirming distinct degradation behaviors of PFOS and PFHxS under identical CAP conditions.

As shown in Fig. 5b, PFHxS degradation follows first-order kinetics both with and without gas dispersion, indicating that the degradation rate remains proportional to the PFHxS concentration in solution. In the absence of gas dispersion, PFHxS degradation followed first-order kinetics with a rate constant of 0.04 1/min (R^2^ = 0.98), corresponding to a half-life of 17.3 min. In contrast, the application of gas dispersion significantly enhanced the degradation rate, increasing the rate constant to 0.17 1/min (R^2^ = 0.99) and reducing the half-life to 4.1 min.

### Degradation products and fluorine mass balance

**Fig. 6:**
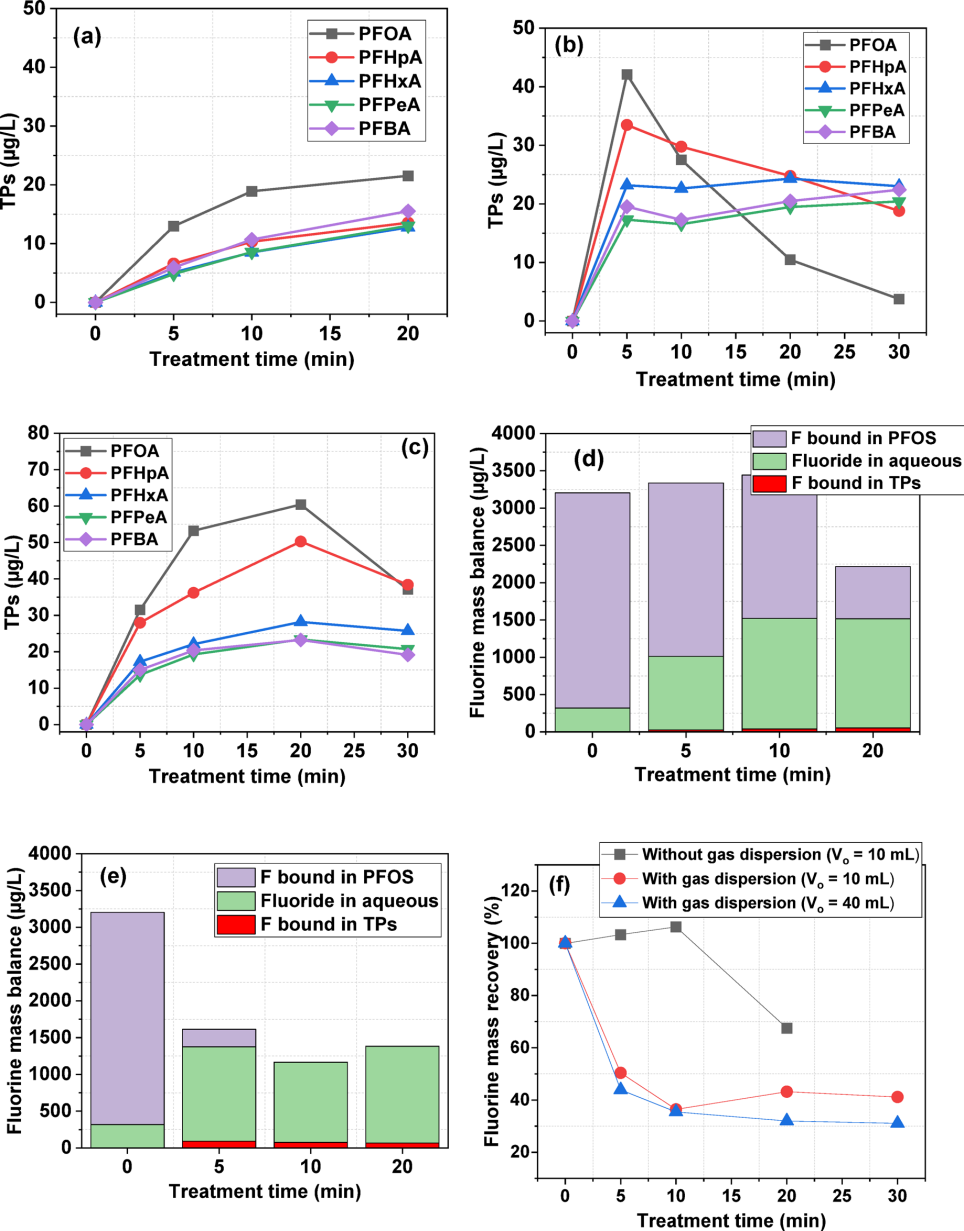
TPs formed during PFOS degradation and fluorine mass balance. (**a**) formation of TPs as a function of treatment time (without gas dispersion and at V₀ = 10 mL). (**b**) TP concentration as a function of treatment time (with gas dispersion and at V₀ = 10 mL). (**c**) TP concentrations over treatment time (with gas dispersion and at V₀ = 40 mL). (**d**) fluorine mass balance (without gas dispersion at and V₀ = 10 mL). (**e**) The fluorine mass balance (with gas dispersion and at V₀ = 10 mL). (**f**) Fluorine mass recovery (%) (at V₀ = 10 mL and 40 mL). The experiments were conducted with a gas-dispersion flow rate of 0.1 L/min and input power of 12 W.

The formation of TPs during PFOS degradation was systematically investigated during CAP treatment. The concentrations of PFOA (C8), PFHpA (C7), PFHxA (C6), PFPeA (C5) and PFBA (C4) were quantified in the µg/L range. Their temporal evolution differed markedly depending on whether gas dispersion was applied, indicating distinct degradation pathways and transport regimes (shown in Fig. 6a–c). In the absence of gas dispersion (shown in Fig. 6a), TP concentrations increased gradually and monotonically over time, consistent with progressive stepwise chain shortening via reductive and oxidative pathways occurring primarily at the static plasma-liquid interface. The slow accumulation of intermediates in the bulk liquid suggests limited mass transfer from the bulk to the interface.

In contrast, gas-dispersed CAP (shown in Fig. 6b) produced a rapid initial increase in longer-chain intermediates, particularly PFOA and PFHpA within the first 5 min, followed by a decline at longer treatment times. This behavior indicates continued transformation of intermediate species into shorter-chain fluorinated products accompanied by partial fluoride release. At larger initial solution volumes with gas dispersion (Fig. 6c), higher absolute TP concentrations were detected; however, the concentrations of all TPs decreased after 20 min, indicating continued degradation and partial defluorination. This decline, even at higher volumes, suggests that bubble-induced mixing efficiently transports intermediates back to the interfacial reactive zone, where they undergo further transformation. The observed concentration trend (PFOA > PFHpA > PFHxA > PFPeA > PFBA) supports a progressive chain-shortening degradation pathway during PFOS treatment. These findings demonstrate that gas dispersion not only accelerates PFOS degradation but also enhances the subsequent transformation of intermediate products through improved plasma-liquid interfacial transport and mixing. Longer treatment durations are necessary to reduce TP concentrations to the desired levels or achieve higher degradation. Future studies will also investigate the performance of the CAP reactor over extended operation times to ensure the higher degradation of TP products.

Figure 6d–f summarizes the fluorine mass balance during PFOS degradation with and without gas dispersion. Total liquid-phase fluorine was calculated as the sum of measured inorganic fluoride and organofluorine associated with PFOS and the identified TPs. Because the analysis relied on targeted HPLC–MS quantification of selected PFAS, additional fluorinated intermediates or products may not have been captured, which may contribute to the incomplete fluorine mass balance. In all operating conditions, aqueous fluoride increased with treatment time, indicating progressive C–F bond transformation and fluoride release, while PFOS-bound fluorine declined. Without gas dispersion (shown in Fig. 6d), most fluorine remained associated with residual PFOS, indicating limited defluorination under static interfacial conditions. In contrast, gas-dispersed CAP (shown in Fig. 6e) produced a pronounced decrease in PFOS-bound fluorine and a substantial increase in fluoride concentration, demonstrating enhanced partial defluorination due to improved plasma-liquid coupling, interfacial reactions, and mass transfer. At larger initial liquid volumes with gas dispersion (Fig. S4, Supplementary Material), fluoride release remained significant but progressed more gradually, consistent with transport limitations and reduced exposure of PFOS and TPs to the reactive plasma-liquid interface.

Despite the enhanced PFOS degradation and fluoride formation observed under gas-dispersed conditions, fluorine mass recovery remained incomplete and was lower than that obtained without gas dispersion. Fluorine mass recovery further highlights the differences between operational modes (shown in Fig. 6f). Under non-dispersed CAP conditions, fluorine recoveries ranged from ~ 68% to 106%. In contrast, gas-dispersed systems showed lower recoveries (~ 31% to 50%), indicating incomplete fluorine accounting within the quantified liquid phase. This incomplete fluorine recovery represents an important limitation of the present study, as it suggests that a significant fraction of fluorine was redistributed into unquantified reservoirs rather than being fully recovered as fluoride or identified fluorinated products in solution. Potential explanations include the formation of unidentified fluorinated intermediates and TPs, partitioning of fluorinated species into the gas phase or foam phase generated during gas dispersion, adsorption onto reactor surfaces, and possible secondary emissions from the plasma-treated system. These pathways were not directly quantified in the present study and therefore remain unresolved. Discussions of incomplete fluorine mass balances have been widely reported in studies of plasma-based PFAS treatment systems and are consistent with our observations^34,40,52^. Further investigation of fluorine partitioning and fate across liquid, gas, and foam phases is needed to establish a more complete fluorine mass balance under gas-dispersed CAP conditions.

### Degradation and partial defluorination mechanisms of PFOS during CAP treatment

**Fig. 7:**
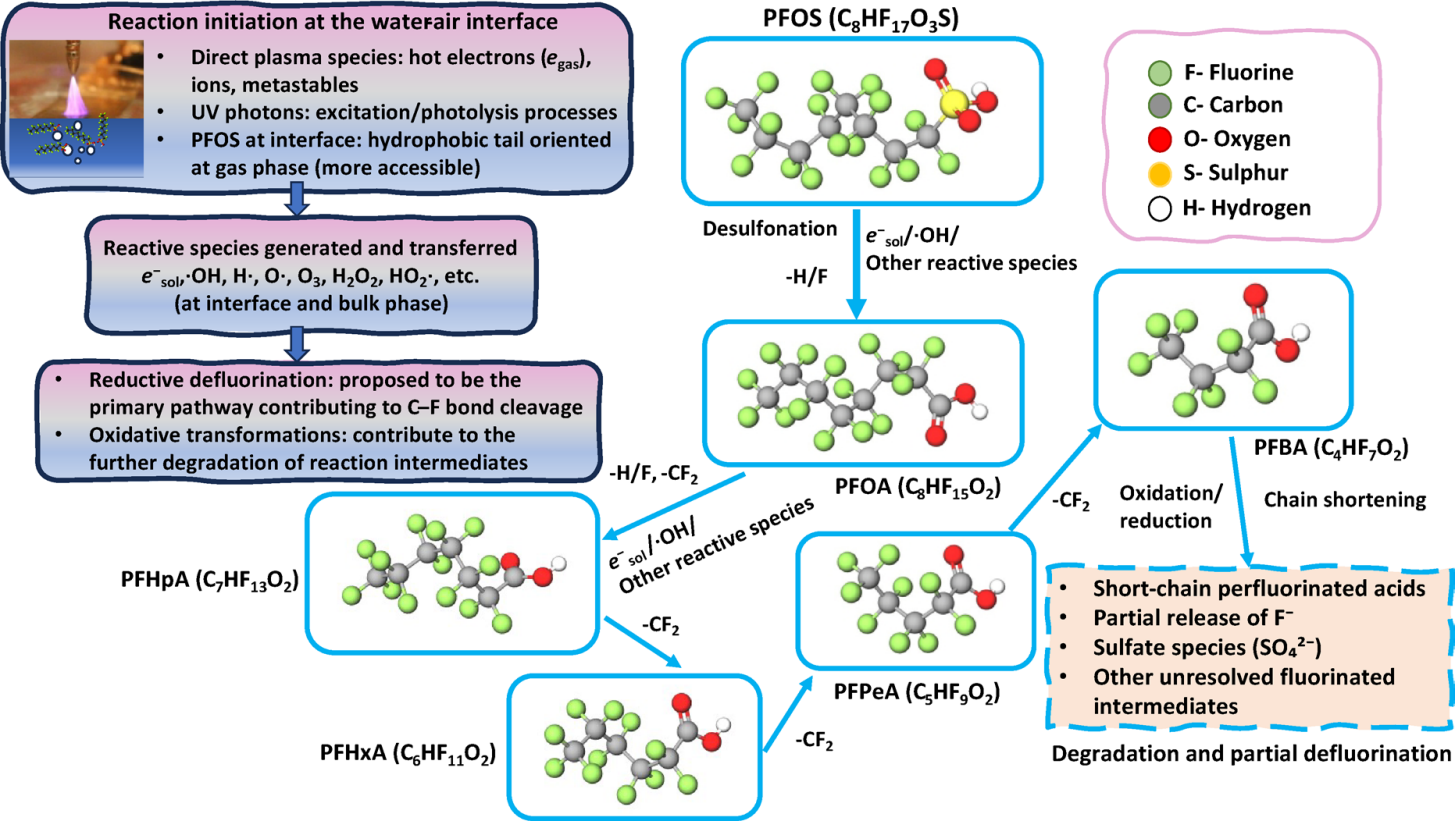
Proposed degradation and partial defluorination pathways of PFOS under CAP treatment. The proposed pathways are based on current understanding of plasma-liquid interfacial chemistry, TPs and fluoride ions released detected in the treated solution. Multiple parallel reductive and oxidative pathways may occur simultaneously. Although several short-chain intermediates were detected, incomplete fluorine recovery indicates that part of the fluorine may be associated with unidentified fluorinated species, gas/foam-phase partitioning, or other unquantified pathways, and its ultimate fate remains unresolved. Experiments were conducted at a gas-dispersion flow rate of 0.1 L/min and input power of 12 W.

The breakdown mechanisms of PFOS under CAP were inferred from the identified TPs and the extent of partial defluorination, which was quantified through fluoride ion measurements. The proposed pathways (Fig. 7) represent plausible dominant routes (although multiple parallel reaction pathways may occur simultaneously) supported by our experimental observations and consistent with some of previous plasma based PFAS treatment studies^4,31,40,65^. CAP generates a complex plasma-chemical environment containing both oxidative and reductive reactive species, produced through electron-impact reactions in the gas phase and subsequently transferred to the plasma-liquid interface and bulk solution through interfacial transport and secondary reaction pathways. The *e*⁻ₛₒₗ are proposed to contribute to PFOS degradation and defluorination^31,38,66^. As strong reductants, *e*⁻ₛₒₗ may participate in electron-transfer reactions with PFOS, potentially contributing to C–F bond cleavage, fluoride release, and the formation of perfluoroalkyl radical intermediates that undergo stepwise chain shortening. Short-lived oxidative species, such as •OH, O•, and others, are not considered the primary agents responsible for PFOS defluorination; however, they may contribute significantly to secondary oxidation reactions and the further transformation of intermediate products, particularly in complex matrices^39^. These reactive species can also participate in the oxidation of the fluorinated sulfonate head group of PFOS, thereby facilitating subsequent fragmentation and degradation pathways in conjunction with interfacial electron-transfer processes^30^.

The proposed mechanistic interpretation is further supported by quenching experiments reported by Chen et al.^40^, in which scavenging *e*⁻ₛₒₗ significantly reduced defluorination efficiency (from ~ 25% to ~ 13%), whereas quenching oxidative radicals had minimal impact. Similar observations were reported by Zhang et al.^39^ during plasma-based PFOA degradation, where nitrate ions (NO_3_⁻) were used as scavengers for *e*⁻ₛₒₗ. Increasing the NaNO_3_ concentration from 0 to 10 mM reduced the PFOA removal efficiency by 44.7%, confirming the strong inhibitory effect of electron scavenging and highlighting the critical role of *e*⁻ₛₒₗ in PFAS degradation. In addition, previous studies have shown that reactive species generated in the gas phase of CAP, as well as plasma-emitted UV radiation, can further contribute in the initial steps of PFAS degradation through direct interfacial interactions^38,66^. PFAS molecules tend to accumulate at the air–water interface due to their surfactant nature, with the hydrophobic fluorinated tail oriented toward the gas phase, making it more accessible to plasma-generated hot electrons, ions, metastables, and photons.

As shown in Fig. 7, possible PFOS degradation under CAP proceeds through a stepwise mechanism involving both reductive and oxidative processes. PFOS degradation is likely initiated by *e*⁻ₛₒₗ attack on the C–S bond, with the first transformation step involving conversion of PFOS to PFOA, which was consistently detected in the treated samples. The reductive cleavage of the C–S bond induces desulfonation through release of the SO_3_⁻ group and formation of the perfluoroalkyl radical (•C_8_F_17_), which is subsequently oxidized to the corresponding carboxylic acid^31,67^. The proposed preference for initial C–S bond transformation over direct C–F bond cleavage is consistent with the higher reactivity of the sulfonate head group and its susceptibility to reductive and oxidative reactions. In contrast, the C–F bonds within the perfluorinated chain possess exceptionally high bond dissociation energies and greater chemical stability, making direct defluorination kinetically less favorable during the initial stages of plasma treatment.

After chain initiation, the newly formed perfluorocarboxylic acids undergo sequential electron-induced reactions and iterative decarboxylation, resulting in progressive shortening of the fluorocarbon chain. This stepwise degradation pathway leads to the formation of shorter-chain perfluorocarboxylic acids, including PFHpA, PFHxA, PFPeA, and PFBA, which were detected as TPs during CAP treatment. These intermediates may undergo further transformation; however, the ultimate fate of carbon- and fluorine-containing species remains unresolved under the present experimental conditions. Importantly, although fluoride ions and multiple short-chain intermediates were detected, the fluorine mass balance remains incomplete, indicating that a portion of fluorine is not accounted for in the quantified liquid-phase species. This missing fluorine may be associated with unidentified fluorinated intermediates or TPs, partitioning of fluorinated species into the gas or foam phases generated during gas dispersion, or possible secondary emissions from the plasma-treatment system.

In the present study, ultra-short-chain PFAS (e.g. trifluoroacetic acid and others), were not detected throughout the treatment, even at LC–MS detection limits as low as 0.05 mol % of the initial PFOS concentration. This absence suggests that transient intermediates were either rapidly transformed, present below the current analytical detection threshold, or partitioned into undetected species. Future work employing more sensitive, time-resolved, and high-resolution analytical techniques will be necessary to establish a more complete fluorine mass balance and further elucidate the mechanistic pathways and ultimate fate of PFOS degradation products under CAP with gas dispersion.

Computational investigations of plasma-water-PFAS interactions, including simulations of electron transport and plasma chemistry, further support the mechanistic interpretations and proposed degradation pathways. For example, Sapunar et al.^68^ employed electron-scattering calculations and potential-energy landscape analyses to examine electron-impact processes associated with PFAS degradation in pulsed dielectric barrier discharge systems. Their plasma model incorporated dissociative excitation, dissociative ionization, dissociative electron attachment, charge-exchange reactions, excitation-transfer pathways, and PFAS decomposition reactions, thereby providing molecular-level insight into electron-driven degradation mechanisms. Song et al.^4^ investigated PFOA degradation using plasma coupled with a fluid dynamics model and a parallel-flow solver to simulate fundamental discharge units. Their simulations resolved electron propagation pathways and electric field distributions at the gas–liquid interface, providing insight into electron penetration into the aqueous phase and the accessibility of reactive electrons to PFOA molecules.

### Energy efficiency analysis of PFOS degradation and partial defluorination

**Fig. 8:**
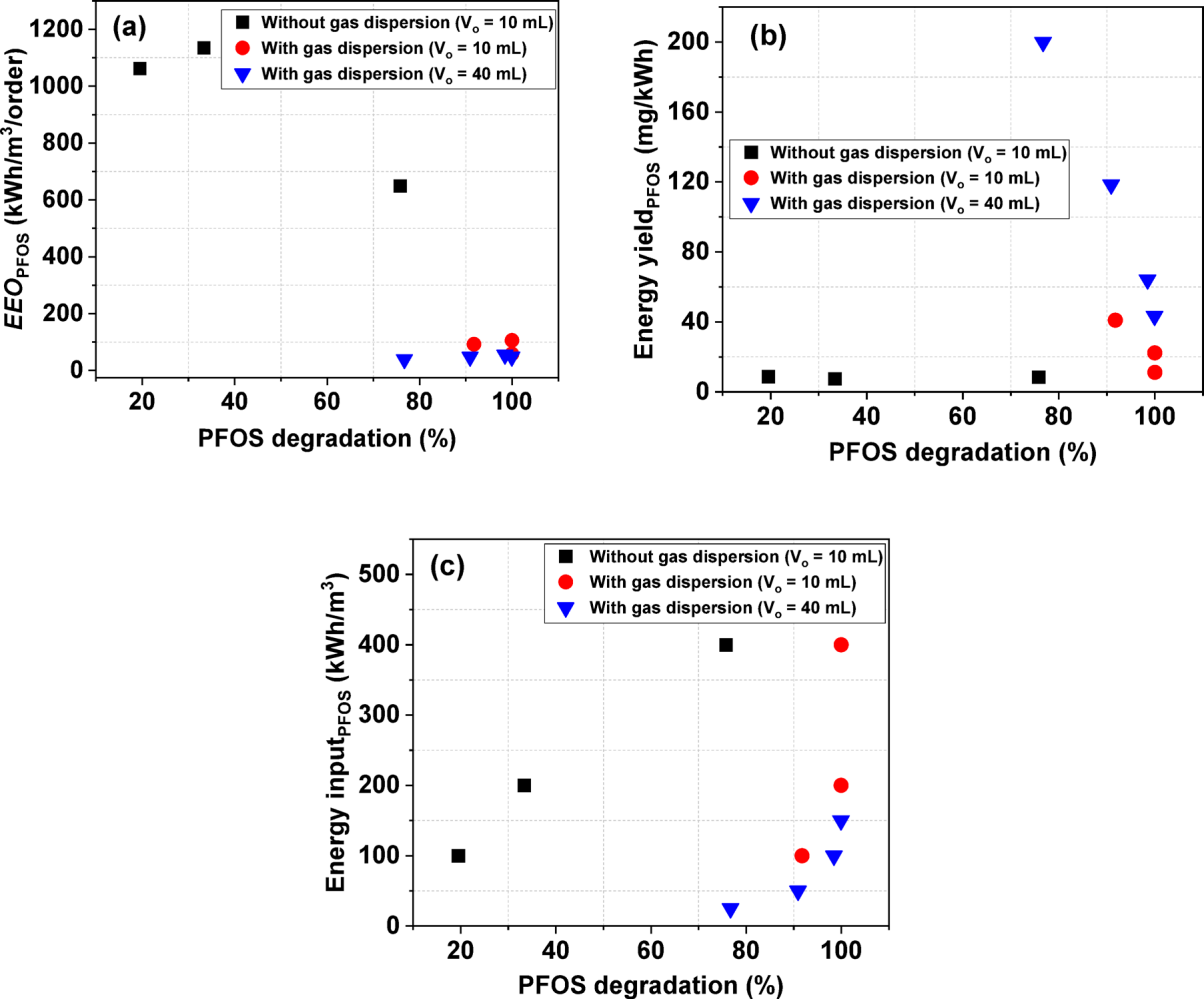
Shows the energy requirements for PFOS degradation and partial defluorination. (**a**) The *EEO* during PFOS degradation. (**b**) The energy yield during PFOS degradation (**c**) energy input. The experiments were conducted at a gas-dispersion flow rate of 0.1 L/min with input power of 12 W.

The CAP treatment system demonstrated a strong capability to efficiently degrade PFOS and shows clear potential for scale-up to large water volumes. Beyond treatment efficiency, energy demand is a critical parameter for assessing the practical feasibility of advanced PFAS destruction technologies. To enable a meaningful comparison across different treatment processes and operational conditions, *EEO* was calculated. The *EEO*, expressed as kWh/m^3^/per log-order reduction, provides a normalized metric that relates energy consumption to contaminant degradation efficiency and is widely used to benchmark AOPs and ARPs^37,69,70^. Figure 8a presents the *EEO* values for PFOS degradation under different CAP operating conditions. Gas dispersion markedly reduced the energy demand of the process, with *EEO* values ranging from 39 to 106 kWh/m^3^/order, achieving rapid PFOS degradation and partial defluorination (35%).

The *EEO* achieved in this study compares favorably with previously reported *EEO* values for PFOS treatment technologies, as shown in Table 2. However, direct comparison studies should be interpreted with caution, as *EEO* is highly dependent on experimental conditions, particularly the initial PFOS concentration. In general, higher initial PFOS concentrations can lead to lower apparent *EEO* values because a greater contaminant mass is degraded per unit energy input, whereas lower concentrations may increase energy demand due to mass-transfer limitations and lower interaction probability with reactive species. In addition, reactor configuration, plasma operating conditions, treatment volume, and water matrix composition can also strongly influence *EEO* values. The comparatively lower *EEO* observed in the present work highlights the important role of coupling CAP with gas dispersion in enhancing plasma-liquid interactions, improving mass transfer, and reducing energy consumption while maintaining efficient PFOS degradation and 35% defluorination. These results demonstrate that gas-dispersed CAP represents a promising, scalable, and energy-efficient strategy for PFAS remediation.

**Table 2:** Comparison of *EEO* values reported in the literature and in the present study for PFOS degradation processes.

Treatment process	PFOS initial concentration (mg/L)	*EEO* (kWh/m^3^/order)	References
CAP + gas dispersion	5	39–106	Present work
Sonochemical	1	502–1644	Ilić et al.^73^
Sonochemical	0.002–5	700–2340	Panda et al.^74^
Sonolysis	10	8000	Moriwaki et al.^75^
Photocatalytic	200	9335	Sharma et al.^76^
Hydrodynamic cavitation	1–5	7–598	Kumar et al.^69^
Direct UV	20	18,190	Yamamoto et al.^77^
UV	18.6	1270	Lyu et al.^78^
Plasma (multi electrodes)	< 0.001–~ 100	380–830	Singh et al.^79^
Gliding arc plasma	100	23.2–213.4	Lewis et al.^37^
Electron Beam	0.1–100	329 ± 53	Londhe et al.^80^
Heat	100	64,447	Yang et al.^81^

Figure 8b presents the energy yield for PFOS degradation, expressed as the mass of PFOS degraded per unit electrical energy input (mg/kWh). The combination of CAP with gas dispersion and larger initial solution volumes resulted in substantially higher energy yields (43–200 mg/kWh), indicating that a greater mass of PFOS was degraded per unit energy input. This improvement reflects more efficient utilization of plasma-generated reactive species and enhanced mass transfer at the plasma-bubble–liquid interface. For comparison, previous plasma-based PFOS treatment studies reported lower energy yields. In the past studies, Yasuoka et al.^71^ achieved 26–80 mg/kWh using argon plasma at an initial PFOS concentration of 17.7 mg/L, while Aziz et al.^72^ reported 22–27 mg/kWh at 1 mg/L (evaluated at 50% conversion). The higher energy yields observed in the present work demonstrate that CAP coupled with gas dispersion enhances the efficiency of electrical energy conversion into PFOS degradation on a mass basis.

The electrical energy input required for PFOS degradation was quantified on a volumetric basis (kWh/m^3^), as shown in Fig. 8c. Reporting energy consumption per unit volume treated is essential for assessing process scalability and practical feasibility in water and wastewater applications. Under gas-dispersed conditions and at higher initial liquid volumes, energy inputs ranged from 25 to 150 kWh/m^3^. Expressing performance in kWh/m^3^ enables direct comparison with alternative destruction technologies. For example, incineration, a conventional thermal method for PFAS destruction typically requires approximately 1312 kWh/m^3^ to heat and vaporize water to temperatures above 1100 °C^82,83^, which is 8–52 times higher than the values observed in present work. The comparatively low energy demand demonstrated in this study highlights the potential of gas dispersion-assisted CAP, which operates under ambient conditions, as an energy-efficient approach for high-volume PFAS remediation, pending further scale-up and process optimization.

### Future research outlook

CAP represents a promising and versatile technology for PFOS degradation and partial defluorination, owing to its ability to generate a broad spectrum of highly reactive oxidative and reductive species that are proposed to contribute to C–F bond cleavage and transformation. Future research should focus on optimizing plasma-liquid hydrodynamics to further enhance PFOS degradation and defluorination. As demonstrated in this work, coupling CAP with gas dispersion markedly accelerates PFOS degradation and partial defluorination by increasing the plasma-liquid interfacial area, inducing bulk mixing and turbulence, and enhancing mass transfer of PFAS from the bulk solution to the reactive interfacial zone. In addition, plasma-bubble interactions promote the formation of higher concentrations of reactive species, further strengthening degradation and defluorination kinetics. Continued refinement of bubble size distribution, gas hold-up, interfacial area density, and recirculation strategies will be critical to maximize interfacial exposure and energy efficiency.

While long-chain PFAS such as PFOS are efficiently transported to the interface due to their strong surface activity, short-chain TPs exhibit weaker interfacial affinity and tend to remain in the bulk liquid. Although gas dispersion partially facilitates TP transport, further improvements in bulk mixing, recirculation strategies, and control of bubble size and distribution are required to enhance their exposure to plasma-generated reactive zones. The use of different working gases (for example, argon, oxygen, nitrogen, or mixed-gas systems) can provide an effective strategy for tailoring plasma chemistry and improving degradation selectivity toward long and short-chain PFAS. In particular, argon-based plasmas can enhance discharge stability because of their lower breakdown voltage and efficient electron-impact excitation processes, thereby promoting the generation of high electron densities and *e*⁻_sol_ at the plasma-liquid interface^31,35^. The addition of argon, either alone or in combination with reactive gases such as oxygen or nitrogen, can further modulate the production of reactive oxygen and nitrogen species, plasma microdischarge characteristics, electron-transfer reactions, and interfacial energy transfer processes. Such plasma-chemical tuning could enhance both PFOS degradation and subsequent defluorination of short-chain transformation products by increasing their exposure to highly reactive interfacial environments.

In addition, the introduction of specific additives, such as cationic surfactants, can promote electrostatic interactions and complexation with negatively charged PFAS TPs, particularly short-chain species, thereby enhancing their accumulation near the gas–liquid and plasma-liquid interfaces^25,38^. In the presence of bubbles, such interactions may facilitate the adsorption or trapping of short-chain PFAS around the bubble surface, increasing their transport to interfacial plasma reaction zones where highly reactive species and microdischarges are concentrated. This interfacial enrichment could improve subsequent degradation and defluorination efficiency of soluble short-chain byproducts that would otherwise remain in the bulk liquid phase. Furthermore, optimization of reactor design and plasma-liquid interaction configurations represents another important avenue for advancement. Improved plasma coupling, discharge uniformity, bubble distribution, and interfacial contact area could enhance reactive species generation, mass transfer, and energy efficiency, thereby enabling more effective PFAS degradation and defluorination.

Future research should also focus on the direct diagnostics and quantification of short-lived reactive species, particularly *e*⁻_sol_ and •OH radicals, at plasma-liquid interfaces. In the present work, only indirect estimation and discussion of these reactive species were provided based on PFOS degradation behavior, fluoride release, H₂O₂ formation, TPs, and established plasma chemistry reported in the literature. However, direct measurement of these species is critically important because they are believed to play major roles in PFOS degradation and defluorination pathways. Advanced diagnostic techniques, including laser-based spectroscopy, time-resolved optical methods, absorption spectroscopy, and selective scavenger-based kinetic approaches, should therefore be explored in future studies to directly quantify reactive species generation and transport under gas-dispersed plasma conditions.

Collectively, the results show that gas dispersion enhances PFOS degradation and partial defluorination; however, incomplete fluorine recovery indicates that complete mineralization cannot be confirmed under the present conditions. The findings suggest fluorine redistribution among aqueous, gaseous, interfacial, and reactor-associated phases. Future work should focus on comprehensive fluorine mass balance, including gas-phase trapping, total organic fluorine (TOF) analysis, fluoride quantification, and extraction of reactor-associated species, together with identification of volatile or condensable fluorinated byproducts. Further studies are also needed to clarify foam formation, plasma–foam interactions, and interfacial chemistry, where fluorinated species may accumulate and contribute to volatilization and transfer to the gas phase. These efforts are essential to resolve fluorine fate and evaluate the potential for complete mineralization under optimized plasma conditions.

Reactor design innovation represents a parallel opportunity for advancement. Configurations such as plasma generation within gas bubbles or across thin falling liquid films could increase effective plasma-liquid interfacial area while improving energy efficiency. Integration of real-time diagnostics to monitor reactive species formation, fluorine release, and intermediate evolution would further enhance mechanistic understanding and process control. Hybrid treatment strategies also warrant attention. Because many PFAS remediation technologies rely on separation processes that concentrate rather than destroy contaminants, coupling CAP with upstream concentration steps can provide an effective treatment-chain approach that minimizes secondary waste. Systematic evaluation of energy efficiency, durability, scalability, and very high defluorination of short-chain byproducts will ultimately determine the viability of CAP for field deployment.

## Conclusions

This study demonstrates the effectiveness of CAP, operated with and without gas dispersion, for the degradation and partial defluorination of concentrated PFOS in tap water. Plasma-liquid interactions generate a complex mixture of reactive species, including *e*⁻_sol_, •OH, and other short-lived species, which are proposed to contribute to PFOS degradation and defluorination. The formation of fluoride ions provides a quantitative indicator of partial defluorination of PFOS. Coupling CAP with gas dispersion markedly enhances treatment performance by enriching PFOS at the plasma-liquid interface, increasing interfacial area, promoting microdischarge formation, inducing bulk mixing, and facilitating the generation and transport of reactive species, all of which collectively promote progressive PFOS degradation and partial defluorination.

Under gas-dispersed conditions, PFOS degradation reached up to 99.99%, with partial defluorination of 35%. Kinetic analysis revealed a transition from zero-order degradation in the absence of gas dispersion (rate constants of 0.17 mg/L/min, corresponding to half-life of 13.4 min) to first-order kinetics upon the introduction of gas dispersion (rate constants of 0.22–0.42 1/min, corresponding to half-life of 1.6–3.0 min), reflecting enhanced mass transfer and more efficient plasma-liquid interfacial reaction dynamics.

Fluorine mass balance analysis showed recoveries ranging from ~ 31–106% across all conditions. Under non-gas dispersed CAP conditions, fluorine recoveries were relatively higher (~ 68–106%), whereas gas-dispersed systems exhibited substantially lower recoveries (~ 31–50%), indicating incomplete fluorine accounting in the aqueous phase. This discrepancy suggests redistribution of fluorine to unidentified fluorinated species, as well as partitioning into gas and/or foam phases and possible secondary emissions from the reactor system. Five TPs were identified, and their concentrations decreased with extended treatment, indicating continued degradation of intermediate species. The observed trend in peak concentrations (PFOA (C8) > PFHpA(C7) > PFHxA(C6) > PFPeA(C5) > PFBA(C4)) suggests stepwise chain-shortening through sequential degradation of PFOS and partial defluorination.

Based on these findings, a degradation mechanism involving initial desulfonation followed by hydro-defluorination, hydroxy-defluorination, chain shortening, and stepwise C–F bond cleavage is proposed. The results are consistent with the hypothesis that plasma-generated reactive species, including both oxidative and reductive species, contribute to PFOS degradation via coupled reaction pathways, resulting in the formation of shorter-chain fluorinated intermediates and partial fluoride release. However, complete mineralization cannot be confirmed due to incomplete fluorine recovery and unresolved fluorine fate under gas-dispersed conditions. Further studies are required to elucidate fluorine redistribution across liquid, gas, foam, and reactor-associated phases and to assess the potential role of secondary emissions under optimized plasma treatment conditions.

Importantly, the *EEO* for PFOS degradation and partial defluorination under gas-dispersed CAP was as low as 39 kWh/m^3^/order, which is substantially lower than many reported values for PFOS treatment technologies. These results highlight the strong potential of gas-assisted CAP as an efficient, chemical-free, and energy-efficient technology for PFAS degradation. Further optimization of reactor design, plasma-liquid hydrodynamics, and operating conditions may enable higher defluorination efficiencies at environmentally relevant concentrations and support scalable real-world applications.

## Supplementary Information

Supplementary Information 1.

Supplementary Video 1.

## Data Availability

All data supporting the findings of this study are available within the paper and its Supplementary Information files. Additional information is available from the corresponding author upon reasonable request.
