# Supplementary material for: Enhanced degradation and defluorination of perfluorooctane sulfonate (PFOS) in tap water using gas-dispersed cold atmospheric plasma
Source: Sci Rep. 2026 Jun 13;16:18363. doi: 10.1038/s41598-026-57490-6 (PMC13264616; doi:10.1038/s41598-026-57490-6)
Supplement: Supplementary file 1 — Supplementary Information 1. [file 41598_2026_57490_MOESM1_ESM.docx]

**Supplementary material**

**Enhanced degradation and defluorination of perfluorooctane sulfonate (PFOS) in tap water using gas-dispersed cold atmospheric plasma**

Amit Kumar^1*^, Ysabel Huaccallo-Aguilar^1^, Holger Kryk^1^, Uwe Hampel^1,2^, Sebastian Felix Reinecke^1^

1. *Clean Water Technology Lab (CLEWATEC), Institute of Fluid Dynamics, Helmholtz Zentrum Dresden-Rossendorf, Bautzner Landstrasse 400, 01328 Dresden, Germany*
2. *Technische Universität Dresden, Chair of Imaging Techniques in Energy and Process Engineering, Dresden, 01062, Germany*

**Corresponding author:** Amit Kumar ([a.kumar@hzdr.de](mailto:a.kumar@hzdr.de))

**Table S1.** Cold atmospheric plasma (CAP) treatment of PFOS with and without gas dispersion (air flow rate 0.1 L/min). Mean values and standard deviations (SD) of HPLC–MS measurements for PFOS, PFHxS, and transformation products (PFOA(C8), PFHpA(C7), PFHxA(C6), PFPeA(C5), PFBA(C4)), expressed in µg/L, are reported to indicate measurement uncertainty. The target initial PFOS concentration was 5 mg/L; however, slight deviations were observed between experimental batches. These differences likely arise from minor losses during sample preparation and handling, including adsorption of PFOS to container surfaces and tubing, as well as pipetting and mixing variability. All calculations in this study were therefore performed using the measured initial PFOS concentrations for each batch.

| \|  \|  \| **PFOS**  **(C8), (µg/L)** \| **SD** \| **PFOA**  **(C8), (µg/L)** \| **SD** \| **PFHpA**  **(C7), (µg/L)** \| **SD** \| **PFHxA**  **(C6), (µg/L)** \| **SD** \| **PFPeA**  **(C5), (µg/L)** \| **SD** \| **PFBA**  **(C4), (µg/L)** \| **SD** \| **PFHxS**  **(C6), (µg/L)** \| **SD** \| \| --- \| --- \| --- \| --- \| --- \| --- \| --- \| --- \| --- \| --- \| --- \| --- \| --- \| --- \| --- \| --- \| \|  \| **Initial PFOS (5 mg/L) in tap water** \| 4465.04 \| 12.50 \| 0 \| 0 \| 0 \| 0 \| 0 \| 0 \| 0 \| 0 \| 0 \| 0 \| 3.97 \| 0.13 \| \| **CAP (without gas dispersion), Vo = 10 mL** \| **5 min treatment** \| 3594.52 \| 39.18 \| 13 \| 0.047 \| 6.6 \| 0.019 \| 5.11 \| 0.052 \| 4.82 \| 0.05 \| 5.93 \| 0.009 \| 3.56 \| 0.133 \| \| **10 min treatment** \| 2975.64 \| 103.6 \| 18.9 \| 0.121 \| 10.3 \| 0.067 \| 8.53 \| 0.059 \| 8.56 \| 0.134 \| 10.7 \| 0.036 \| 3.31 \| 0.007 \| \| **20 min treatment** \| 1081.31 \| 8.215 \| 21.5 \| 0.166 \| 13.5 \| 0.278 \| 12.8 \| 0.31 \| 13 \| 0.551 \| 15.5 \| 0.028 \| 2.8 \| 0.107 \| \| **CAP (with gas dispersion), Vo = 10 mL** \| **5 min treatment** \| 368.20 \| 3.643 \| 42.1 \| 0.072 \| 33.5 \| 0.335 \| 23.2 \| 0.462 \| 17.3 \| 0.164 \| 19.5 \| 0.199 \| 3.11 \| 0.0208 \| \| **10 min treatment** \| 1.68 \| 0.004 \| 27.5 \| 0.154 \| 29.8 \| 0.0268 \| 22.6 \| 0.108 \| 16.5 \| 0.049 \| 17.3 \| 0.18 \| 2.4 \| 0.0074 \| \| **20 min treatment** \| 0.75 \| 0.013 \| 10.5 \| 0.149 \| 24.8 \| 0.0992 \| 24.3 \| 0.795 \| 19.5 \| 0.534 \| 20.5 \| 0.111 \| 1.54 \| 0.0061 \| \| **30 min treatment** \| 0.03 \| 0.003 \| 3.75 \| 0.080 \| 18.8 \| 0.0226 \| 23 \| 0.124 \| 20.4 \| 0.124 \| 22.4 \| 0.020 \| 1.21 \| 0.0152 \| \|  \| **Initial PFOS (5 mg/L) in tap water** \| 6516 \| 20.2 \| 0 \| 0 \| 0 \| 0 \| 0 \| 0 \| 0 \| 0 \| 0 \| 0 \| 4.37 \| 0.16 \| \| **CAP (with gas dispersion), Vo = 40 mL** \| **5 min treatment** \| 1517 \| 6.98 \| 31.5 \| 0.022 \| 28 \| 0.042 \| 17.3 \| 0.071 \| 13.6 \| 0.211 \| 15 \| 0.033 \| 3.6 \| 0.006 \| \| **10 min treatment** \| 593 \| 2.43 \| 53.2 \| 0.234 \| 36.2 \| 0.062 \| 22.1 \| 0.02 \| 19.2 \| 0.035 \| 20.4 \| 0.027 \| 3.77 \| 0.025 \| \| **20 min treatment** \| 101 \| 1.4 \| 60.4 \| 0.085 \| 50.3 \| 0.075 \| 28.02 \| 0.048 \| 23.4 \| 0.293 \| 23.3 \| 0.564 \| 3.77 \| 0.071 \| \| **30 min treatment** \| 4.86 \| 0 \| 37.1 \| 0.045 \| 38.4 \| 0.096 \| 25.7 \| 0.013 \| 20.7 \| 0.277 \| 19.1 \| 0.308 \| 3 \| 0.065 \| |
| --- | --- | --- | --- | --- | --- | --- | --- | --- | --- | --- | --- | --- | --- | --- | --- | --- | --- | --- | --- | --- | --- | --- | --- | --- | --- | --- | --- | --- | --- | --- | --- | --- | --- | --- | --- | --- | --- | --- | --- | --- | --- | --- | --- | --- | --- | --- | --- | --- | --- | --- | --- | --- | --- | --- | --- | --- | --- | --- | --- | --- | --- | --- | --- | --- | --- | --- | --- | --- | --- | --- | --- | --- | --- | --- | --- | --- | --- | --- | --- | --- | --- | --- | --- | --- | --- | --- | --- | --- | --- | --- | --- | --- | --- | --- | --- | --- | --- | --- | --- | --- | --- | --- | --- | --- | --- | --- | --- | --- | --- | --- | --- | --- | --- | --- | --- | --- | --- | --- | --- | --- | --- | --- | --- | --- | --- | --- | --- | --- | --- | --- | --- | --- | --- | --- | --- | --- | --- | --- | --- | --- | --- | --- | --- | --- | --- | --- | --- | --- | --- | --- | --- | --- | --- | --- | --- | --- | --- | --- | --- | --- | --- | --- | --- | --- | --- | --- | --- | --- | --- | --- | --- | --- | --- | --- | --- | --- | --- | --- | --- | --- | --- | --- | --- | --- | --- | --- | --- | --- | --- | --- | --- | --- | --- | --- | --- | --- | --- | --- | --- | --- | --- | --- | --- | --- | --- | --- | --- | --- | --- | --- | --- | --- | --- | --- | --- | --- |

**Fig. S1.** Change in PFOS concentration as a function of treatment time: **(a)** with and without gas dispersion (V₀ = 10 mL) and **(b)** with gas dispersion (V₀ = 40 mL). The error bars are small, indicating high reproducibility and precision of the analytical measurements. The experiments were conducted at a gas-dispersion flow rate of 0.1 L/min and an input power of 12 W.

**Fig. S2.** PFOS degradation and partial defluorination over treatment time (V₀ = 40 mL). The experiments were conducted at a gas-dispersion flow rate of 0.1 L/min and input power of 12 W.

**Fig. S3.** First-order kinetics with gas dispersion (V₀ = 40 mL). All experiments were conducted at a gas-dispersion flow rate of 0.1 L/min and an input power of 12 W.

**Fig. S4.** The fluorine mass balance (with gas dispersion and at V₀ = 40 mL).

**Text S1: Mathematical equations (part of material and methods)**

**PFOS degradation efficiency**

[PFOS]_o_​ and [PFOS]_t_ are the PFOS concentrations (mg/L) at time 0 and time 𝑡, respectively. This metric represents the fraction of PFOS removed from solution during treatment.

$$\text{PFOS degradation, \% = }\frac{\text{[PFOS]}_{\text{o}}\text{- }\text{[PFOS]}_{\text{t}}}{\text{[PFOS]}_{\text{o}}}\text{ ×100}$$

**Defluorination efficiency**

Defluorination was used to quantify C–F bond cleavage and the release of fluoride ions. It represents the fraction of fluorine originally bound in PFOS that is converted to inorganic fluoride (F⁻) during treatment and thus reflects the extent of PFOS mineralization. Here, [TF]_o_ denotes the initial total fluorine content (mol/L) present in PFOS.

$$\text{Defluorination, \%= }\frac{{\text{[}\text{F}^{\text{-}}\text{]}}_{\text{t}}\text{- }{\text{[}\text{F}^{\text{-}}\text{]}}_{\text{o}}}{\text{[TF]}_{\text{o}}}\text{×100}$$

**Fluorine mass recovery**

Fluorine mass recovery quantifies the fraction of total fluorine accounted for at time *t* by summing the measured inorganic fluoride [F^−^]_t_ ​ and the estimated fluorine remaining in PFOS and its transformation products [TF]_t_, relative to the initial total fluorine content. This metric assesses fluorine mass balance by accounting for both released and residual fluorine, and helps identify potential fluorine losses to unmeasured phases, such as volatilization or adsorption onto reactor surfaces.

$$\text{Fluorine }\text{mass recovery, \%= }\frac{{\text{[}\text{F}^{\text{-}}\text{]}}_{\text{t}}\text{ + }\text{[TF]}_{\text{t}}}{{\text{[}\text{F}^{\text{-}}\text{]}}_{\text{o}}\text{ + }\text{[TF]}_{\text{o}}}\text{×100}$$

**Reaction kinetics**

PFOS degradation kinetics were evaluated by monitoring its concentration as a function of treatment time. The data were fitted to kinetic models that provided the best agreement based on the regression coefficient (R²): a first-order model for gas-dispersed CAP conditions and a zero-order model for treatments without gas dispersion.

$$\text{First order kinetics (k), 1/min= }\frac{\text{[PFOS]}_{\text{t}}}{\text{[}\text{PFOS]}_{\text{o}}}\text{= }\text{e}^{\text{-k}\text{t}}$$

The first-order half-life was measured to calculate the time required for 50% degradation of PFOS:

$$\text{[t]}_{\text{1/2 }}\text{,min = }\frac{\text{0.693}}{\text{k}}$$

The zero-order rate (*k* in mg/L/min) equation is:

$$\text{ }\text{[PFOS]}_{\text{t }}\text{= -kt + }\text{[PFOS]}_{\text{o }}\text{ }$$

The half-life (min) for a zero-order reaction is given by:

$$\text{[t]}_{\text{1/2 }}\text{ = }\frac{\text{[PFOS]}_{\text{o}}}{\text{2k}}$$

**Electrical energy per order (*EEO*)**

The *EEO*, a log-reduction-based efficiency metric, was calculated to quantify the energy required to reduce the PFOS concentration by one order of magnitude in one cubic meter of water. This metric enables direct comparison of energy efficiency across different treatment processes, system configurations, and operational scales.

*V*_o_ is the treated volume (m^3^), and [PFOS]_o_ and [PFOS]_t_ represents the initial concentration and the concentration at time *t* (mg/L), respectively.

$$\text{EEO in kWh/}\text{m}^{\text{3}}\text{/order= }\frac{\text{Input power}\text{ }\left( \text{kW} \right)\text{ × treatment time (h)}}{\text{V}_{\text{o}}\text{ (}\text{m}^{\text{3}}\text{) ×}\text{log}_{\text{10}} \text{(}\frac{\text{[PFOS]}_{\text{o}}}{\text{[PFOS]}_{\text{t}}}\text{)}}$$

**Energy yield**

Energy yield, expressed in mg/kWh, is a mass-based removal efficiency metric that quantifies the amount of PFAS, specifically PFOS, removed per unit of energy consumed. It enables comparison of the efficiency of treatment systems in terms of contaminant mass removal relative to energy input. A higher energy yield indicates a more efficient process, potentially lowering operational costs for contaminant treatment. It should be noted that, the energy yield is highly dependent on the initial concentration of the target compound. The energy yield was calculated as follows:

$$\text{ Energy yield,}\frac{\text{mg}}{\text{kWh}}\text{=}\frac{\text{[PFOS]}_{\text{o}}\text{ (}\frac{\text{mg}}{\text{L}}\text{) × }\text{V}_{\text{o}}\text{ }\left( \text{L} \right)\text{ × PFOS degradation \% ×}\frac{\text{1}}{\text{100}}\text{ }}{\text{Input power }\left( \text{kW} \right)\text{ × treatment time (h)}}$$

**Energy input**

Energy input, expressed in kWh/m³, is a volume-based cost metric that quantifies the total electrical energy required to treat one cubic meter of water. It provides an estimate of the overall treatment cost per unit volume and serves as a basis for preliminary benchmarking of the treatment process. The energy input was calculated as follows:

$$\text{ Energy input,}\frac{\text{kWh}}{\text{m}^{\text{3}}}\text{= }\frac{\text{Input power }\left( \text{kW} \right)\text{ × treatment time (h) }}{\text{V}_{\text{o}}\text{ }\left( \text{m}^{\text{3}} \right)}$$

**Estimation of solvated electron concentration**

The concentration of solvated electrons (*e*⁻_sol_) was estimated indirectly from the decay of PFOS assuming pseudo-first-order kinetics. PFOS was treated as the dominant scavenger of solvated electrons, with a reported second-order rate constant *k*_1_ = 1 × 10^9^ M^-1^s^-1^. The reaction is.

PFOS + *e*⁻_sol_ → ​​products

If the steady-state concentration of solvated electrons remains approximately constant during treatment, the PFOS decay follows:

$$\frac{\text{[PFOS]}_{\text{t}}}{\text{[}\text{PFOS]}_{\text{o}}}\text{= }\text{e}^{\text{-k}_{\text{obs}}\text{t }}$$

Where, *k*_obs_ is the observed first-order rate constant, defined as:

$$\text{ }\text{k}_{\text{obs}}\text{= }\text{k}_{\text{1}}\left[ \text{e}_{\text{sol}}^{\text{-}} \right]$$

Thus, the solvated electron concentration is obtained from:

$$\left[ \text{e}_{\text{sol}}^{\text{-}} \right]\text{= }\frac{\text{1}}{\text{k}_{\text{1}}\text{t}}\text{ln(}\frac{\text{[PFOS]}_{\text{o}}}{\text{[}\text{PFOS]}_{\text{t}}}\text{)}$$

The resulting values are reported in µmol/L.

This approach assumes PFOS is the primary scavenger of solvated electrons and neglects competing reactions with other reactive species, which may reduce the apparent *e*⁻_sol_ ​ concentration. Mass-transfer limitations between the plasma, bubble phase, and bulk liquid were not explicitly included. Therefore, the calculated concentrations represent order-of-magnitude estimates specific to the experimental CAP conditions.

**Calculation of air bubble number**

Air was introduced into the liquid through a 4 mm pipe at a flow rate of 0.1 L/min. Assuming spherical bubbles with a diameter (d) of 4 mm, the number of bubbles generated per minute was calculated as follows:

$$\text{Volume of one bubble,}\text{ml}\text{= }\frac{\text{π }}{\text{6}}\text{ × }\text{d}^{\text{3}}$$

$$\text{Number of bubbles,}\text{N}_{\text{bubbles}}\text{, 1/min= }\frac{\text{Air flow rate (mL/min) }}{\text{Volume of one bubble (ml)}}$$
